# Two RNA recognition motif-containing proteins are plant mitochondrial editing factors

**DOI:** 10.1093/nar/gkv245

**Published:** 2015-03-23

**Authors:** Xiaowen Shi, Maureen R. Hanson, Stéphane Bentolila

**Affiliations:** Department of Molecular Biology and Genetics, Cornell University, Ithaca, NY 14853, USA

## Abstract

Post-transcriptional C-to-U RNA editing occurs in plant plastid and mitochondrial transcripts. Members of the *Arabidopsis* RNA-editing factor interacting protein (RIP) family and ORRM1 (Organelle RNA Recognition Motif-containing protein 1) have been recently characterized as essential components of the chloroplast RNA editing apparatus. ORRM1 belongs to a distinct clade of RNA Recognition Motif (RRM)-containing proteins, most of which are predicted to be organelle-targeted. Here we report the identification of two proteins, ORRM2 (organelle RRM protein 2) and ORRM3 (organelle RRM protein 3), as the first members of the ORRM clade to be identified as mitochondrial editing factors. Transient silencing of *ORRM2* and *ORRM3* resulted in reduced editing efficiency at ∼6% of the mitochondrial C targets. In addition to an RRM domain at the N terminus, ORRM3 carries a glycine-rich domain at the C terminus. The N-terminal RRM domain by itself provides the editing activity of ORRM3. In yeast-two hybrid assays, ORRM3 interacts with RIP1, ORRM2 and with itself. Transient silencing of *ORRM2* in the *orrm3* mutant further impairs the editing activity at sites controlled by both ORRM2 and ORRM3. Identification of the effect of ORRM2 and ORRM3 on RNA editing reveals a previously undescribed role of RRM-containing proteins as mitochondrial RNA editing factors.

## INTRODUCTION

In land plants, C-to-U RNA editing is a post-transcriptional modification that occurs in plastids and mitochondrial transcripts, often resulting in changes in the amino acid sequence from what the genomic sequence predicts. In *Arabidopsis*, 43C targets are modified in plastids whereas over 600 Cs are edited in mitochondria (1–3). The amino acid encoded by edited transcripts is usually more conserved than the one predicted by unedited transcripts (4). The current concept of RNA editing is that it is essential for correction of defective transcripts that would otherwise affect the proper function of gene products (5,6).

The composition of the RNA editosome is not yet fully understood although *cis-* as well as *trans-*factors have been found to be essential for the editing process. *cis*-elements that specify the editing of the C target are known to be present in close proximity to the editable C (7–9). Members of the PLS subclass of the pentatricopeptide repeat (PPR) motif-containing family are site-specific recognition factors required for RNA editing in plant organelles (10,11). A code has been proposed to explain the specific recognition of *cis*-elements by PPR proteins (12–14). While the cytidine deaminase catalyzing C-to-U conversion has not been identified by expression of a protein with enzymatic activity, considerable evidence points to the C-terminal DYW domain found on some PPR proteins, which exhibits sequence similarity to known cytidine deaminase motifs (15). Mutagenesis of conserved deaminase residues in DYW1, QED1 and RARE1 resulted in loss of editing activity (16,17), further strengthening the hypothesis that the enzyme activity resides in the DYW domain.

Members of other plant protein families have also been identified as *trans-*factors in the RNA editosome. RIP1 (RNA-editing factor interacting protein (1)) is a major editing factor that controls editing at hundreds of sites in plastids and mitochondria (18). Other members of RIP/MORF family are also plastid or mitochondrial editing factors (1,19). The chloroplast editing factor ORRM1 (Organelle RNA Recognition Motif-containing protein 1) was discovered through a database search with the RIP1 protein sequence (20). ORRM1 contains a truncated RIP-RIP domain at its N terminus and an RRM domain (RNA Recognition Motif) at its C terminus. The RRM domain by itself is able to provide chloroplast RNA editing activity to *orrm1* mutants (20). ORRM1 belongs to a distinct clade of RRM-containing proteins, most of which are predicted to be organelle-targeted (20).

In order to identify additional components of the RNA editing apparatus and to further characterize the role of the ORRM family in editing, we investigated the function of the ORRM clade through analysis of mutant and silenced tissues. Expression of two members of the clade, encoded by At5g54580 and At5g61030, were found to affect editing at many mitochondrial sites. We named them ORRM2 (organelle RRM protein 2) and ORRM3 (organelle RRM protein 3), respectively. Transient silencing of *ORRM2* resulted in reduction of editing efficiency at 35 mitochondrial sites, whereas the transient silencing of *ORRM3* caused decrease of editing extent at 32 mitochondrial sites. The editing defects were also observed in an *orrm3* mutant. Transgenic expression of the *ORRM3* coding region restored editing extent at sites affected in the mutant. Thus, different members of the ORRM clade are required for either plastid (ORRM1) or mitochondrial (ORRM2, ORRM3) RNA editing. Our identification of the first ORRM family members as mitochondrial editing factors provides significant new information concerning the composition of plant mitochondrial editosomes. Members of three different protein families—the PPR protein family, the RIP/MORF protein family and now the ORRM protein family—are all required for plant mitochondrial RNA editing. Furthermore, our results establish the ORRM clade as an important group from which additional RRM editing factor proteins may be detected in the future.

## MATERIALS AND METHODS

### Plant material

The Arabidopsis T-DNA insertion line SALK_038244 in the *ORRM3* gene was ordered from the Arabidopsis Biological Resource Center (https://abrc.osu.edu/). After 3 days of stratification, seeds were planted in soil growing in a growth room (14 h of light/10 h of dark) at 26°C. Genotyping was done by polymerase chain reaction (PCR) with Bioline BioMix Red using primer pair SALK_038244-LP and SALK_038244-RP listed in Supplementary Table S1. The PCR product was sequenced at Cornell University Life Sciences Core Laboratories Center. Leaves were collected from 5-week old plants for further analysis.

### Constructs used in this study

In order to perform Virus Induced Gene Silencing (VIGS), primers flanking a gene-specific region were selected from the CATMA database (21). Fragments were amplified from *Arabidopsis* Columbia ecotype genomic DNA using primer pairs ORRM2-vigs-F/ORRM2-vigs-R and ORRM3-vigs-F/ORRM3-vigs-R respectively. The fragment was first integrated into PCR8/GW/TOPO vector (Invitrogen) and then shuffled into the silencing vector PTRV2/GW/GFP in a LR Clonase II (Invitrogen) recombination reaction. cDNA clones of ORRM2 and ORRM3 used in this study were reverse-transcribed by SuperScript^®^ III Reverse Transcriptase (Life Technologies) from RNA extracted from wild-type *Arabidopsis* Columbia using PureLink^®^ RNA Mini Kit (Life Technologies). Primers used are listed in Supplementary Table S1. For complementation experiments, full-length *ORRM3* coding sequence was amplified using primer pair ORRM3-F and ORRM3-R from the reverse-transcribed cDNA and then cloned into PCR8/GW/TOPO vector. The fragment was transferred to pAUL1 vector (22) by LR reaction. N-terminal RRM domain of ORRM3 was amplified from the reverse-transcribed cDNA using primers ORRM3-F and ORRM3–360R, cloned into PCR8/GW/TOPO vector first and then a modified PBI121 vector using LR Clonase II. For yeast two-hybrid (Y2H) assays, mature *ORRM2* coding sequence, without the sequence for the predicted 34 aa transit peptide, was amplified with primer pair ORRM2–103F and ORRM2-R from the reverse-transcribed cDNA, while mature *ORRM3* coding sequence that lacks the predicted 36 aa transit peptide sequence was amplified with primer pair ORRM3–109F and ORRM3-R. PCR products were ligated to PCR8/GW/TOPO vector and then shuffled to pGADT7GW and pGBKT7GW vectors (23) respectively. RIP1, RIP3 and MEF1 constructs were described previously (18).

### Virus-induced gene silencing

Silencing constructs carrying either *ORRM2* or *ORRM3* fragment were transformed into *Agrobacterium tumefaciens* GV3101, which were used to infiltrate 2-week old *Arabidopsis* seedlings expressing 35S-GFP as previously described (18). Green Fluorescent Protein (GFP) was used as a marker to track silencing efficiency 2 weeks after infiltration. Leaves from *GFP*-silenced plants, which exhibited dark red chlorophyll fluorescence, but no green fluorescence, under UV light were collected for further analysis. To transiently silence *ORRM2* in *orrm3* mutants, 2-week old *orrm3* mutant seedlings were infiltrated with Agrobacterium carrying a *GFP/ORRM2* co-silencing construct. *orrm3* mutants that were not infiltrated and *orrm3* mutants infiltrated with Agrobacterium carrying a *GFP-*silencing construct were used as controls. The silencing efficiency of *ORRM2* was measured by quantitative RT-PCR.

### Use of strand-and transcript-specific RT-PCRseq (STS-PCRseq) method to assay editing extent

The STS-PCRseq technique has been presented in detail in a previous paper (1). Briefly, the method consists in amplifying all transcripts encoding either mitochondrial or chloroplast genes from the RNA extracted from the silenced or the control plants. The RT-PCR amplicons obtained with organelle-specific primers were mixed in equimolar ratio for each plant in order to achieve the same read depth for every transcript. After shearing of the cDNA mix by sonication, the sample was used as a template for the production of an Illumina TruSeq DNA Library. We pooled all the samples described in this report in one sequencing lane of an Illumina HiSeq 2500 instrument. The processing of the reads and their alignments parameters followed the guidelines we established previously (1). The C ≥ T editing sites were determined for each sample using the likelihood ratio test with error rates computed empirically from alignment data, as described in detail in a protocol ‘Identification of CT editing sites’ (1).

### Identification of organelle editing sites exhibiting a reduced editing extent in *ORRM*-silenced plants

The difference in editing extent (T/C + T) between each control plant, not inoculated or GFP-silenced and an *ORRM*-silenced plant at a given editing site was tested by a chi-square test with one degree of freedom. The number of C reads and T reads were pooled together between biological replicates for the control plants. Because of repetitive testing we adjusted the nominal error rate by a Bonferroni correction in order to achieve the desired familywise error rate (*P* < 1e-3). In the case of 618 mitochondrial sites this adjustment results in an error rate of *P* < 1.6e-6; while for the 38 plastid sites the nominal error rate was *P* < 2.6e-5. In addition to the chi-square test requirement, a site was declared significantly reduced in its editing extent if the reduction compared to the control plant was ≥0.1.

Fifty four mitochondrial sites and three plastid sites exhibited a significant reduction of editing extent in the *GFP*-silenced control when compared to the uninoculated plants, because virus inoculation can induce a non-specific effect on editing extent (1). These sites were therefore not included in the analysis of plants silenced for *ORRM2* and *ORRM3*. Sites significantly reduced in the *ORRM*-silenced plants versus uninoculated plants were further checked against the *GFP*-silenced control. The only sites that were declared to be significantly reduced in their editing extent were those sites that exhibited a significant reduction in *ORRM*-silenced plants when compared with both control plants, uninoculated and *GFP*-silenced.

### Generation of transgenic plants

35S-ORRM3 in the pAUL1 vector was transformed into *A. tumefaciens* GV3101 and floral dip transformation of homozygous *orrm3–1* mutants (SALK_038244) was performed as described in (24). Plants were selected on soil by spraying Basta twice. The transgene was verified by PCR using primer pair ORRM3-F and HA-R, while the homozygosity of mutant allele was validated by PCR with SALK_038244-F and SALK_038244-R. Leaves from 4-week old transgenic plants were collected for further analysis. 35S-nORRM3 in PIB121 vector was transformed and selected in the same way, except that the primers used were PBI121-F and ORRM3–360R.

### Measures of editing extent (other than STS-PCRseq)

RNA was extracted using PureLink^®^ RNA Mini Kit (Life Technologies), cleared from contaminating trace amounts of DNA by using a turbo DNA-free kit (Ambion), followed by reverse transcription with SuperScript^®^ III Reverse Transcriptase (Life Technologies). Primers used are listed in Supplementary Table S1 or provided in previous studies (1,18,25). Editing extent was examined by either bulk Sanger sequencing or Poisoned Primer Extension (PPE) assay as described previously (26,27).

### Y2H assay

Two mating types of yeast strain PJ69–4, a and α, were transformed as described in (28). Double transformants were obtained by mating single transformants of different mating type. Afterward, double transformants were cultured in leucine- and tryptophan-deficient media and then diluted with sterile water to 1 × 10^6^, 1 × 10^5^ cells/ml. Ten microliters of each dilution was spotted on leucine-, tryptophan-, adenine- and histidine-deficient media plates. Empty vectors were used as negative control to test autoactivation. Data was collected from 2 d to 10 d after spotting.

### Real-time quantitative RT-PCR conditions and analysis

Quantification of RNA was performed with a nanodrop spectrophotometer (Nanodrop Technologies) or with a Qubit 2.0 fluorometer (Invitrogen) using the Qubit RNA BR assay kit (Molecular Probes). cDNA was reverse transcribed from RNA with primers listed in Supplementary Table S1. Real-time PCRs were followed with a Bio-Rad CFX Connect Real-Time PCR detection system using iTaq Universal SYBR Green Supermix (Bio-Rad). Reactions were initiated by incubating the samples at 95°C for 3 min to activate Taq polymerase, followed by 40 cycles of 10 s at 95°C and 30 s at 55°C. Melting-curve analysis was performed starting at 65°C with stepwise temperature elevations of 0.5°C every 5 s to check for non-specific products. PCR primer sequences used to amplify the different genes assayed are listed in Supplementary Table S1. Reactions contained 7.5 μl of 2× SYBR iTaq Universal SYBR Green Supermix (Bio-Rad), 5 μl of cDNA (0.2 ng μl − 1) and 250 nm of each product-specific primer in a final volume of 15 μl. Data were analyzed using the CFX Manager Software system (Bio-Rad). The results of the quantitative RT-PCR analysis were normalized using three genes, At2g28390, At4g34270 and At5g25760, which have been shown to be superior reference genes for transcript normalization in Arabidopsis (29).

## RESULTS

### Transient silencing of *ORRM2* and *ORRM3* leads to mitochondrial editing defects

In order to characterize the function of the ORRM family proteins in RNA editing, we performed VIGS to transiently silence two *Arabidopsis* genes whose RRMs are closely related to the RRM found in ORRM1 a known plastid editing factor (Supplementary Figure S1). A GFP co-silencing marker was used in the silencing construct to monitor the silencing efficiency (26). Two-week-old *Arabidopsis* seedlings expressing GFP under a 35S promoter were inoculated with Agrobacterium strain carrying either the *GFP* silencing construct as a negative control, the *ORRM2/GFP* co-silencing construct or the *ORRM3/GFP* co-silencing construct. No macroscopic phenotypic defect was observed in either *ORRM2*-silenced or *ORRM3*-silenced plants. Editing extents in RNA from 5-week-old *ORRM*-silenced plants, *GFP*-silenced plants and uninoculated plants were analyzed by a strand- and transcript-specific RNA-seq method (STS-PCRseq) (1).

For each sample, we scanned all the 8320 C sites on the genomic templates to identify sites where the number of T bases in aligned reads was found to be statistically significant. The statistical significance was assessed using a likelihood ratio test comparing how well the observed alignment can be explained by assuming the absence of an edit and assuming an edit with certain proportion of T. The test used empirical mismatch rates calculated for each library from alignments. Details on this calculation and on the statistical test itself have been given previously (1). Using this procedure we identified 656 editing sites in the filtered data, among which 38 are plastid and 618 are mitochondrial (Supplementary Dataset S1). The two biological replicates assayed for each sample were made from cDNA obtained from different plants grown in the same condition. The variability of editing extent between these biological replicates is negligible as demonstrated by the very high correlation found for each pairwise comparison (Supplementary Figure S2).

**Figure 1.**
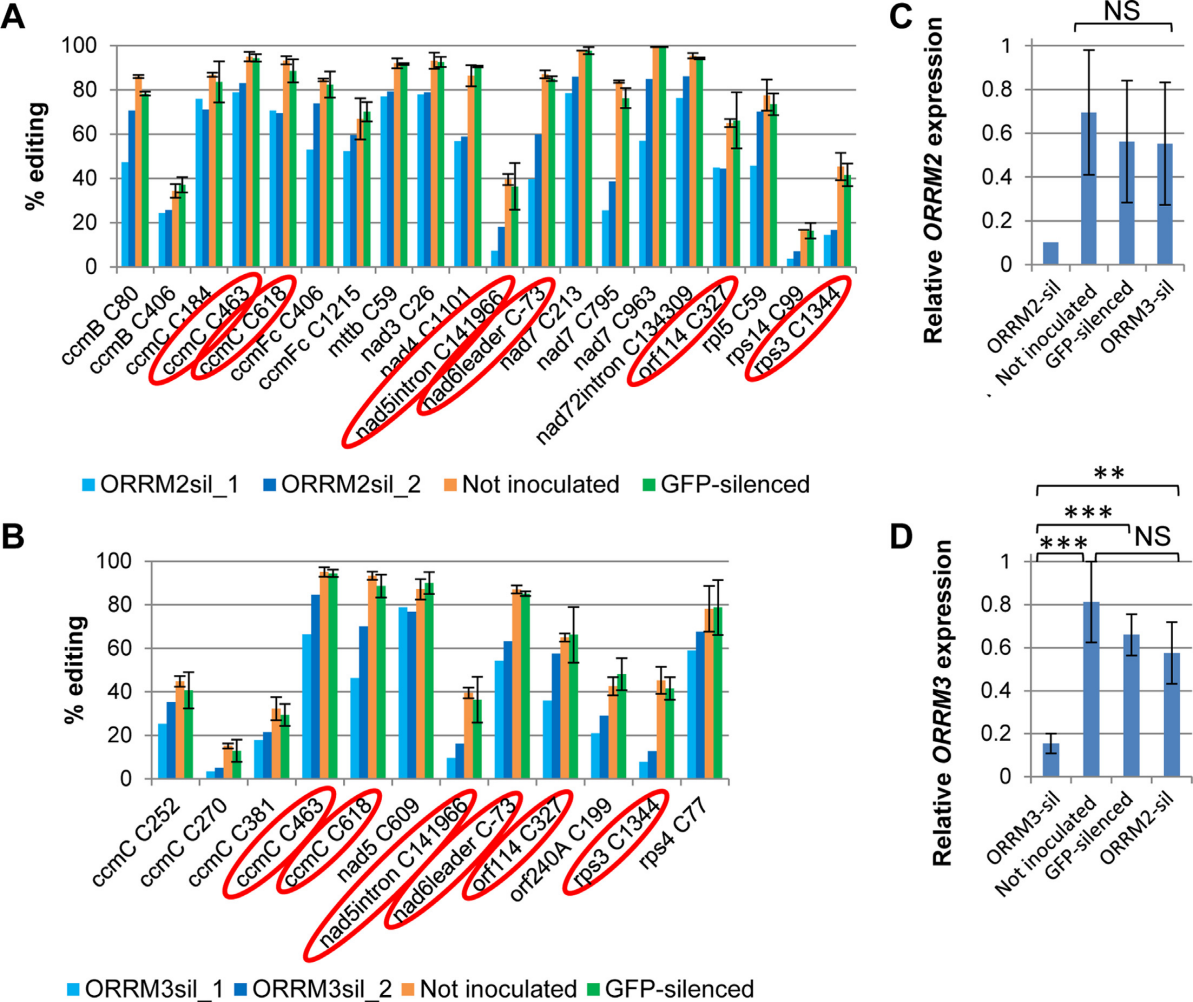
Transient silencing of *ORRM2* and *ORRM3* in *Arabidopsis* results in mitochondrial editing defects. Editing extent was measured by STS-PCRseq. ORRM2-sil, plants inoculated with Agrobacteria harboring a *GFP* and *ORRM2* co-silencing construct. ORRM3-sil, plants inoculated with Agrobacteria harboring a *GFP* and *ORRM3* co-silencing construct. Not inoculated, plants not inoculated with Agrobacteria. *GFP*-silenced, plants inoculated with Agrobacteria harboring a *GFP* silencing construct. Two replicates were used in each silencing experiment. Editing sites that experienced a decrease in editing extent ≥10% between the uninoculated controls and one of the silenced plants are shown. (**A**) Mitochondrial sites showing a reduction of editing extent in *ORRM2-*silenced plants. (**B**) Mitochondrial sites exhibiting a reduction of editing extent in *ORRM3-*silenced plants. Circledare the sites showing reduced editing extent in both silencing experiments. (**C** and **D**) Expression of silenced genes is measured by qRT-PCR. (C) Relative expression level of *ORRM2* is reduced in *ORRM2*-silenced plants. (D) Relative expression level of *ORRM3* is reduced in *ORRM3*-silenced plants. Significance ** (*P* < 0.01), *** (*P* < 0.001), NS (*P* ≥ 0.05).

Transient silencing of *ORRM2* results in a significant reduction (Δ ≥ 10%, *P* < 1.6e-6) of editing extent at 35 mitochondrial sites, while transient silencing of *ORRM3* leads to a significant reduction in editing at 32 mitochondrial sites (Supplementary Tables S2 and S3, Figure 1A, B). Both *ORRM2* and *ORRM3*-silenced plants exhibit a similar proportion of sites with reduced editing extent, 6% of the total of the mitochondrial sites assayed. Interestingly, a rather large proportion, 37–40%, of mitochondrial sites reduced in their editing extent when one of the *ORRMs* is silenced, also exhibits a decreased editing extent when the other *ORRM* is silenced (Supplementary Tables S2 and S3, Figure 1A, B). We did not observe any chloroplast editing defects in these silenced plants (Supplementary Tables S4 and S5). Utilizing a green fluorescent protein assay, a previous report demonstrated that ORRM2 and ORRM3 are localized to mitochondria (30).

To demonstrate the specificity of the silencing experiment, the RNA expression level of *ORRM2* and *ORRM3* was measured by quantitative RT-PCR. The expression level of ORRM2 is knocked down to about 10% in *ORRM2*-silenced tissues compared with the uninoculated and *GFP*-silenced plants (Figure 1C). The expression of *ORRM3* is reduced to about 15% in *ORRM3*-silenced tissues compared with the controls (*P* < 0.001) (Figure 1D).

**Figure 2.**
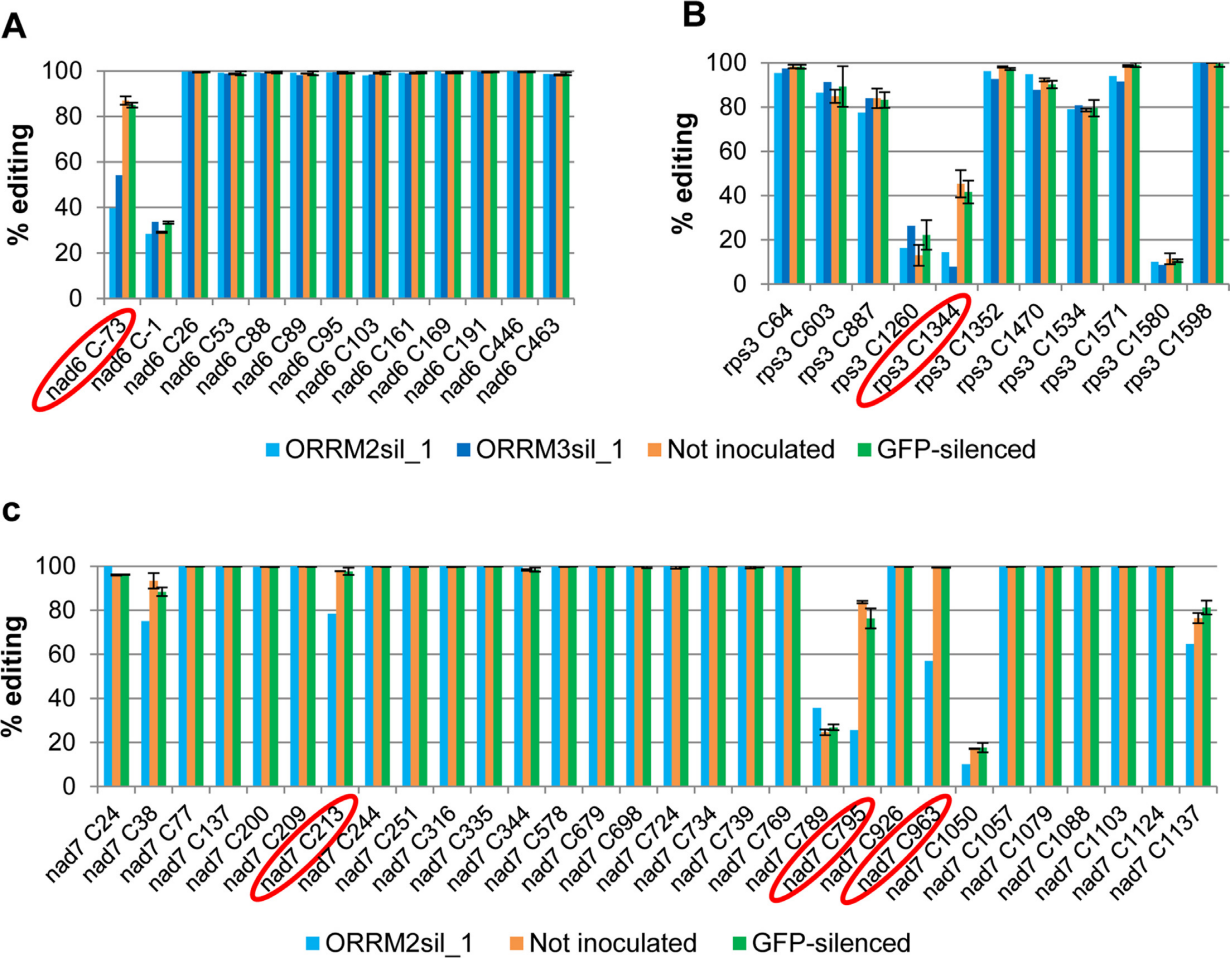
Mitochondrial editing defects in *ORRM*-silenced plants are site-specific. Editing extent was measured by STS-PCR seq. ORRM2-sil, plants inoculated with Agrobacteria harboring a *GFP* and *ORRM2* co-silencing construct. ORRM3-sil, plants inoculated with Agrobacteria harboring a *GFP* and *ORRM3* co-silencing construct. Not inoculated, plants not inoculated with Agrobacteria. *GFP*-silenced, plants inoculated with Agrobacteria harboring a *GFP* silencing construct. Circled are the sites showing a significant reduction of editing extent in the *ORRM*-silenced plants. (**A**) Editing extent of sites on the *nad6* transcript. (**B**) Editing extent of sites on the *rps3* transcript. (**C**) Editing extent of sites on the *nad7* transcript.

The effect of silencing *ORRM2* or *ORRM3* on editing extent is site specific and not transcript specific; only one site is affected on *nad6* and *rps3* transcripts while the other sites do not exhibit any change (Figure 2A and B). Even when several sites on the same transcript show a significant reduction of editing extent in the silenced tissues, these sites are not clustered together and are separated by invariant sites (Figure 2C).

### *ORRM3* mutation leads to reduction of mitochondrial editing

**Figure 3.**
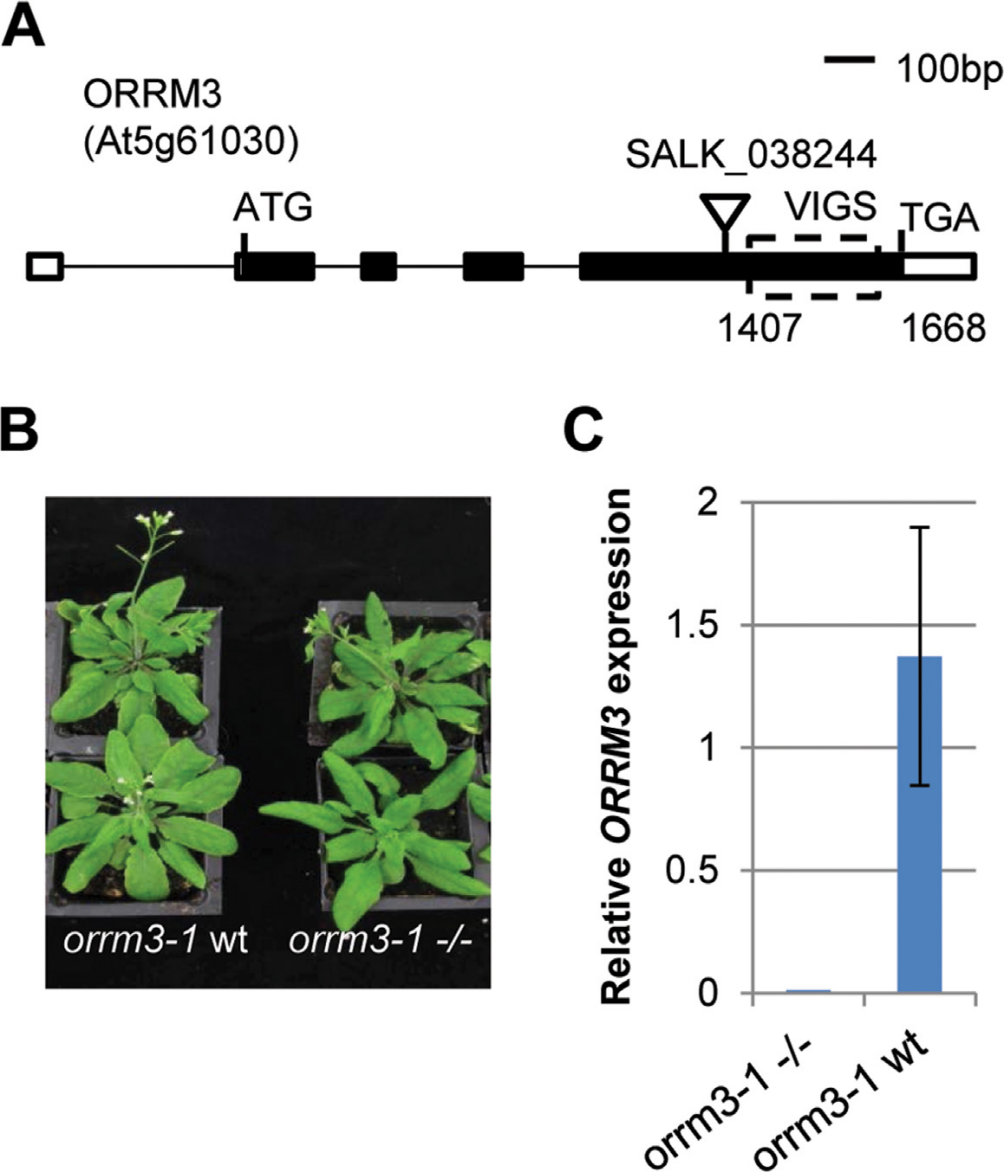
Gene structure, mutant phenotype and mRNA expression level of *ORRM3* (**A**) Gene structure of *ORRM3*. Triangle indicates the location of the T-DNA insertion. Dashed box shows the gene specific region selected for VIGS (Virus Induced Gene Silencing). (**B**) Plant growth phenotype of *orrm3–1* homozygous mutant (right) and its wild-type siblings (left). (**C**) Relative *ORRM3* expression level by quantitative RT-PCR. *ORRM3* expression is not detectable in *orrm3–1* homozygous mutant (left) compared with its wild-type siblings (right).

**Figure 4.**
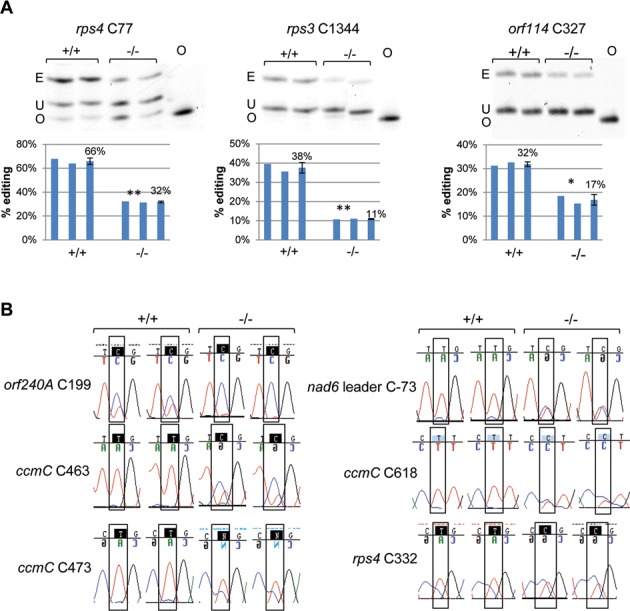
RNA editing at multiple mitochondrial sites is defective in the *orrm3–1* mutant. (**A**) Editing at site *rps4* C77, *rps3* C1344 and *orf114* C327 is significantly reduced in the *orrm3–1* mutant as shown by PPE assay. +/+, wild-type sibling of *orrm3–1*; −/−, homozygous *orrm3–1*. E, edited band; U, unedited band; O, oligonucleotide. Average for each group is displayed in a third bar. Significance * (*P* < 0.05), ** (*P* < 0.01). (**B**) Editing at site *orf240A* C199, *nad6* leader C-73, *ccmC* C463, *ccmC* C618, *ccmC* C473 and *rps4* C332 is disrupted in *orrm3–1* as shown by bulk sequencing RT-PCR products. Portions of electrophoretograms from bulk sequencing are shown. Editable Cs are shown in white letter surrounded by black squares. +/+, wild-type sibling of *orrm3–1*; −/−, homozygous *orrm3–1*.

To further confirm the result of our VIGS experiment, we obtained a T-DNA insertion line in the *ORRM3* gene from ABRC, SALK_038244, in the Columbia background (Figure 3A). The homozygous mutant does not show any obvious morphological defect compared with its wild-type sibling (Figure 3B). *ORRM3* expression is decreased to undetectable level in the mutant measured by quantitative RT-PCR demonstrating that this mutant is a knockout (Figure 3C). We performed PPE and bulk sequencing assays to examine the editing extents of sites affected by the knockout of *ORRM3* (Figure 4). PPE assays demonstrated that the editing extent at site *rps4* C77 decreases from 66 to 32% (*P* < 0.01), site *rps3* C1344 drops from 38 to 11% (*P* < 0.01) and *orf114* C327 editing extent decreases from 32 to 17% (*P* < 0.05) (Figure 4A). According to bulk sequencing, decreases in editing extent are also observed at sites *orf240A* C199, *nad6* leader C-73, *ccmC* C463 and *ccmC* C618 (Figure 4B). The reduction of editing extent in sites examined in *orrm3–1* mutant plants all agrees with the results obtained from the silencing experiment; however, more sites are affected in *orrm3* mutant plants than in *ORRM3*-silenced plants. For instance, editing extent at sites *rps4* C332 and *ccmC* C473 is not significantly reduced in *ORRM3*-silenced plants, but a significant decrease is observed in *orrm3–1* mutant plants (Supplementary Table S3, Figure 4B). This result can be explained by the absence of *ORRM3* expression in *orrm3* mutant plants while *ORRM3* is still expressed in *ORRM3*-silenced plants (Figures 1D and 3C).

### Stable expression of *ORRM3* in *orrm3–1* mutant plants complements the editing defects

**Figure 5.**
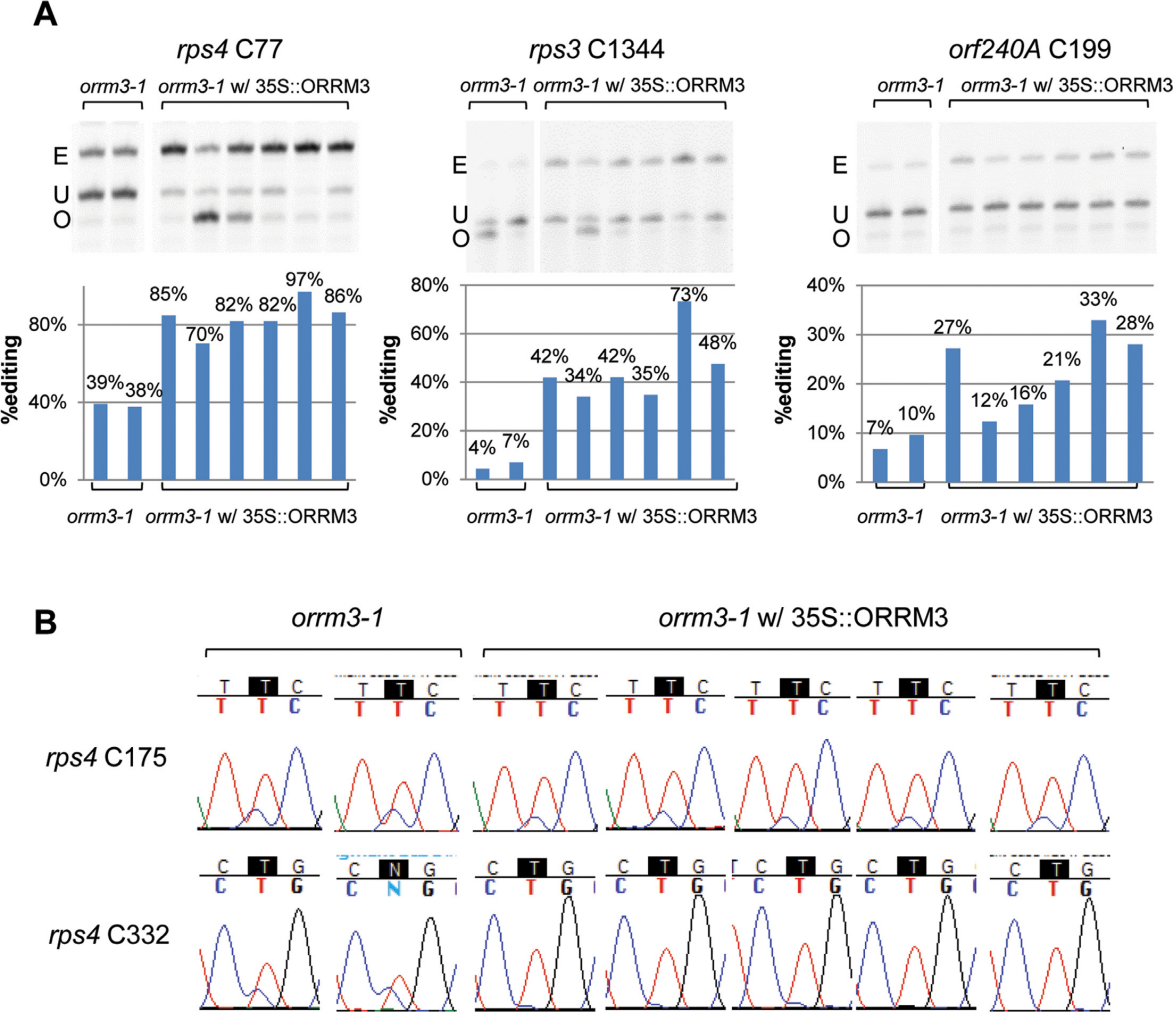
Stable expression of *ORRM3* under a 35S promoter in *orrm3–1* mutant plants complements the editing defects. *orrm3–1*, homozygous *orrm3–1* mutant plants; *orrm3–1* w/ 35S::ORRM3, *orrm3–1* mutant plants transformed with a construct expressing *ORRM3* driven by a 35S promoter.(**A**) Several transgenic mutant plants were assayed by PPE assay. E, edited band; U, unedited band; O, oligonucleotide. (**B**) Editing measured by bulk sequencing shows the complementation to be specific. Portion of electrophoretograms from RT-PCR bulk sequencing is shown. The editable C is shown in white letter in black background. Only the sites being affected in *orrm3–1* mutant plants are complemented by stable expression of ORRM3 (bottom lane), whereas the sites not affected in *orrm3–1* mutant are not complemented (upper lane).

To further prove that a decrease or absence of *ORRM3* expression is the actual cause of editing defects, we transformed homozygous *orrm3* mutant plants with a construct expressing *ORRM3* under the control of a 35S promoter. We performed genotyping to verify that plants surviving the Basta selection are homozygous for the *orrm3* T-DNA insertion allele while carrying the *ORRM3* transgene. All the transgenic plants exhibited a normal phenotype. The editing extents of several independent transgenic plants were analyzed by PPE and bulk sequencing assays. Compared to the homozygous mutants without the transgene, all the complemented plants expressing *ORRM3* showed restoration of editing (Figure 5). The variation of editing extents in different complemented plants is likely a reflection of differential expression of transgenes due to positional effect. For instance, at site *rps4* C77, the editing extent varies from 70 to 97% (Figure 5A), while the wild-type editing level at site *rps4* C77 is about 70% (Figure 4A). Thus, *rps4* C77 editing in most transgenic plants is restored to the wild-type level or even higher.

The same observation can be made for *rps3* C1344; its editing extent in transgenic plants far exceeds the non-transgenic *orrm3* plants with a range from 34 to 73% versus 4–7% (Figure 5A). Some of the transgenic plants also show an editing extent which is higher than the wild-type, 42–-73% versus 38% respectively (Figures 4A and 5A). The small difference in editing extent of the *orrm3–1* mutant plants between Figures 4A and 5A at sites *rps3* C1344 and *rps4* C77, 11 versus 5.5% and 32 versus 38.5% likely results from subtle differences in the timing of the tissue harvest or in the growth conditions of the plants. Editing at site *orf240A* C199 is also complemented (Figure 5A).

The increase of editing extent in complemented lines appears to be site-specific; sites whose editing extent is not affected by a decreased or absent expression of *ORRM3* do not experience an increase in editing extent. For example, the editing of *rps4* C175 is not affected in the *orrm3* mutant or *ORRM3*-silenced plants, accordingly its editing extent is also not modified in *orrm3* transgenic lines (Figure 5B). On the contrary, the editing extent of *rps4* C332 is reduced in the *orrm3* mutant and the defect is complemented in *orrm3* transgenic lines (Figures 4B and 5B). The restoration of editing defects by introduction of a functional *ORRM3* confirms that *ORRM3* encodes a mitochondrial editing factor in *Arabidopsis*.

### ORRM3 interacts with RIP1 in yeast two-hybrid assays

**Figure 6.**
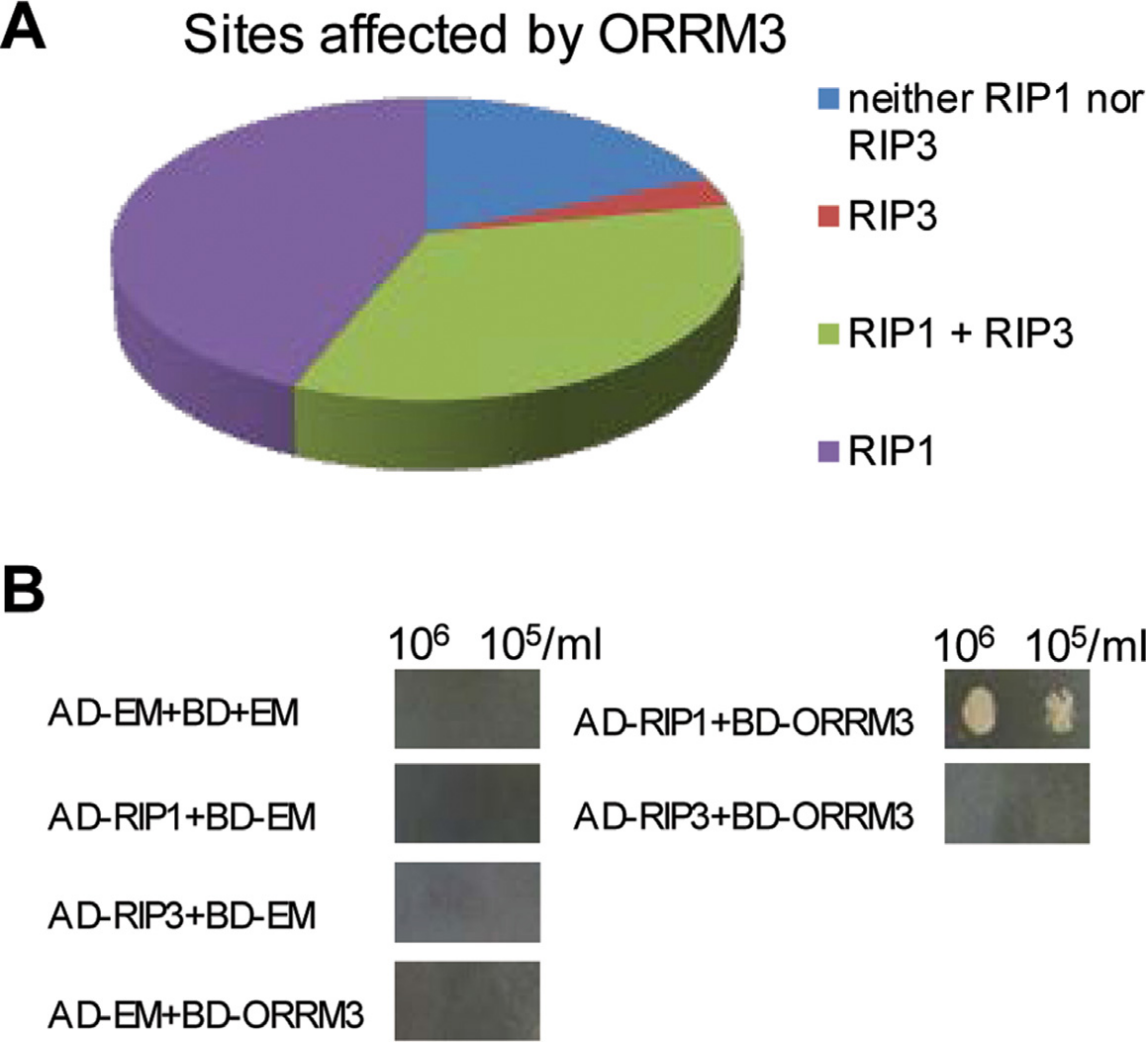
Genetic and physical interaction between ORRM3 and RIPs. (**A**) Distribution of the effect of *RIP1* and *RIP3* mutations on the sites controlled by ORRM3. (**B**) ORRM3 interacts with RIP1 but not RIP3. EM, yeasts transformed with a vector carrying an empty GW cassette as a negative control.

To further characterize the role of ORRM3 in RNA editing, we performed Y2H analysis to test the interaction between ORRM3 and other known mitochondrial editing factors. Among the 32 editing sites affected in *ORRM3*-silenced plants, 25 sites or 78% are also affected in *rip1* mutant plants while 12 sites or 37% are affected in *rip3* mutant plants, implying that ORRM3 may interact with RIP1 and RIP3 in mitochondrial editing (Supplementary Table S6, Figure 6A). To test this hypothesis, we fused the predicted mature coding sequence (removing the predicted transient peptides) to the AD or BD domain. As shown in Figure 6B, BD-ORRM3 interacts with AD-RIP1 but not AD-RIP3 in yeast. The reciprocal mating pairs AD-ORRM3/BD-RIP1 and AD-ORRM3/BD-RIP3 were not tested due to the auto-activation of BD-RIP1 and BD-RIP3. The fact that ORRM3 interacts with RIP1 in yeast further supports the role of ORRM3 as a mitochondrial editing factor.

We also performed Y2H assays to examine the interaction between ORRM2 and RIP proteins. No interaction was observed between ORRM2/RIP1 and ORRM2/RIP3 in yeast (Supplementary Figure S3A). Additionally, we tested the interaction of ORRM2 with MEF1, a PPR recognition *trans* factor whose mutant has been reported to lack detectable editing at *nad7* C963 (31), a site showing a reduced editing extent in *ORRM2*-silenced plants (Figure 1A). ORRM2 did not interact with MEF1, as shown in Supplementary Figure S3B. The absence of identified PPR factors involved in the editing of sites affected in *ORRM3*-silenced plants prevented us from performing a similar interaction experiment with ORRM3.

### ORRM3 interacts with ORRM2 and with itself

**Figure 7.**
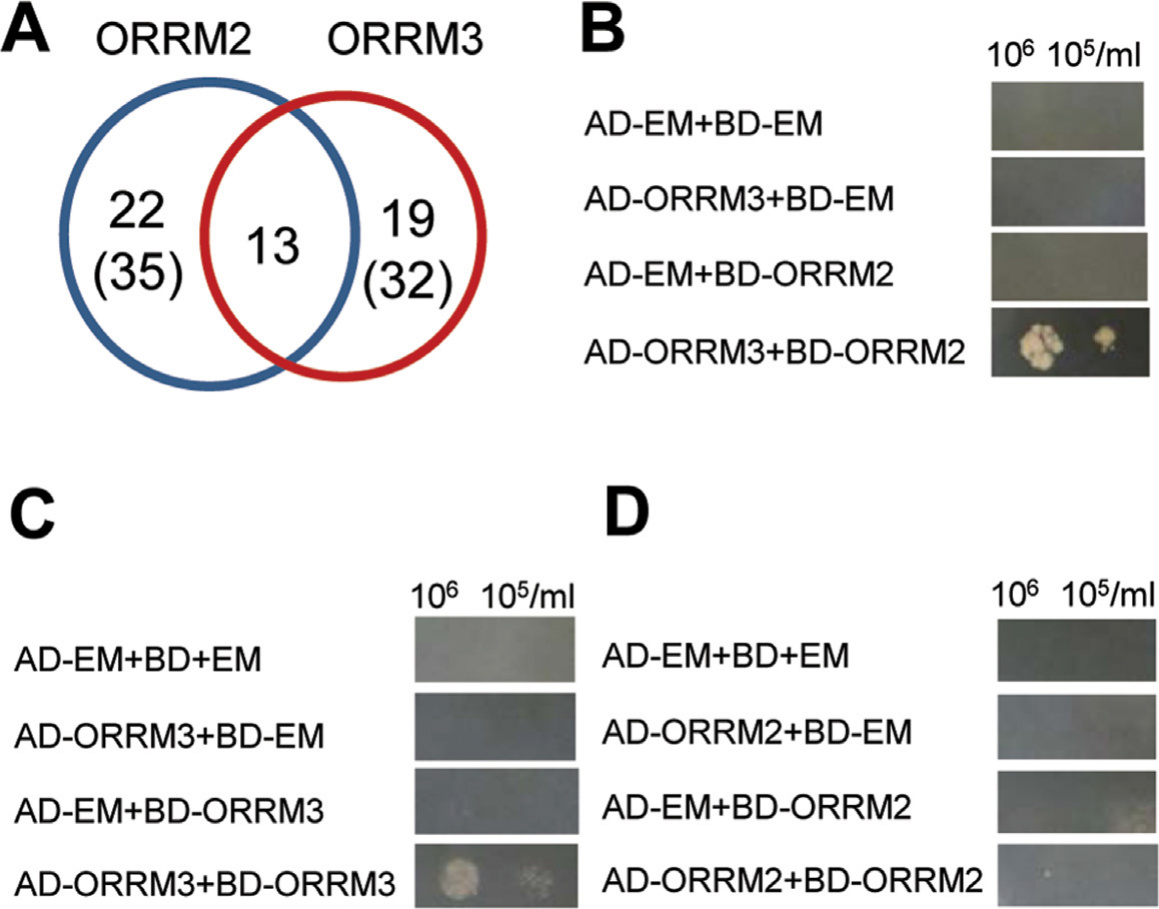
Genetic and physical interaction between ORRM2 and ORRM3. (**A**) Number of mitochondrial sites affected by the transient silencing of *ORRM2* and *ORRM3*. Overlap region indicates the sites under the control of both. Numbers in parentheses refer to the total number of sites affected by the transient silencing of each *ORRM*. (**B**) ORRM3 and ORRM2 form heterodimers. (**C** and **D**) ORRM3 forms homodimer while ORRM2 does not.

When we compared the editing sites affected in *ORRM2*-silenced plants and *ORRM3*-silenced plants, we found a high rate of overlap (37–40%) between these sites (Supplementary Tables S2 and S3, Figure 7A). To test the hypothesis that ORRM2 interacts with ORRM3, we performed Y2H assays and found an interaction between AD-ORRM3 and BD-ORRM2 (Figure 7B). The reciprocal mating between AD-ORRM2 and BD-ORRM3 is not informative due to the auto-activation of BD-ORRM3. Y2H assays also demonstrate that ORRM3 can dimerize in yeast but ORRM2 cannot (Figure 7C and D).

### Transient silencing of *ORRM2* in *orrm3* mutants further impairs the editing efficiency

**Figure 8.**
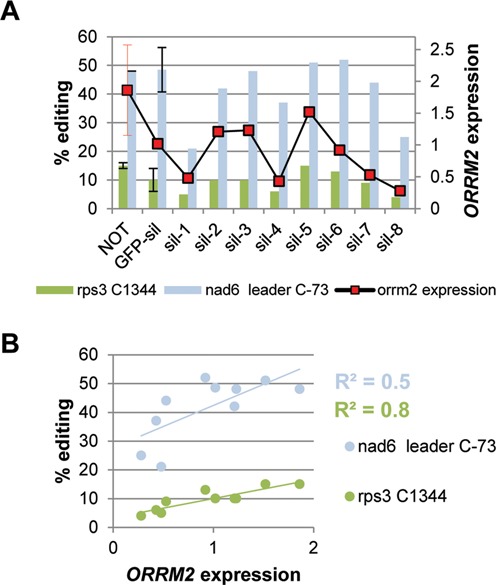
Residual extent in *orrm3* mutant plants can be reduced by silencing *ORRM2*. (**A**) Juxtaposition of editing extent at two sites under the control of ORRM2 and ORRM3 in *ORRM2*-silenced *orrm3* mutant plants and *ORRM2* expression. A solid black line withsquares shows the relative *ORRM2* expression in *orrm3* mutant plants inoculated with Agrobacteria harboring a *GFP* and *ORRM2* co-silencing construct and the controls. Bars refer to the editing extent at sites *rps3* C1344 and *nad6* leader C-73, measured by PPE assay. NOT, *orrm3* mutant plants that were not inoculated with Agrobacteria; GFP-sil, *orrm3* mutant plants inoculated with Agrobacteria harboring a *GFP* silencing construct; sil-1 to sil-8, *orrm3* mutant plants inoculated with Agrobacteria harboring a *GFP* and *ORRM2* co-silencing construct. (**B**) Editing extent in *ORRM2*-silenced *orrm3* mutant plants correlates with the expression of *ORRM2*.

Given that ORRM2 interacts with ORRM3 in yeast, and that both factors control a rather large proportion of common target sites, we wondered whether they were also working synergistically *in vivo*. The lack of a T-DNA insertional mutant for *ORRM2* prevented us from creating a double mutant, so we instead performed VIGS to transiently silence *ORRM2* in *orrm3* mutants. Two-week-old *orrm3* mutant seedlings were inoculated with Agrobacterium strain carrying either the *GFP* silencing construct as a negative control or the *ORRM2/GFP* co-silencing construct (named sil-1 to sil-8 in Figure 8A). The relative expression level of *ORRM2* in different plants was measured by quantitative RT-PCR, while the editing extent was examined by PPE assays. As shown in Figure 8, at sites *rps3* C1344 and *nad6* leader C-73, sites that are controlled by both ORRM2 and ORRM3, the variation of editing extent correlates well with the variation of *ORRM2* expression level, especially for *rps3* C1344 (Figure 8B). The expression of *ORRM3* was not monitored in this experiment because we have shown previously that it is at an undetectable level in *orrm3* mutant plants. The reduction of editing extent is more pronounced in plants with lower *ORRM2* expression. For instance, the editing extent for both *rps3* C1344 and *nad6* leader C-73 is at its lowest for plants sil-1 and sil-8 which also exhibit the lowest (or close to the lowest for plant sil-1) level of *ORRM2* expression (Figure 8A). The reduction of editing extent in plants sil-1 and sil-8 is significant compared to the uninoculated control plants for *rps3* C1344 (*P* < 0.01), while for *nad6* leader C-73 the reduction is significant when compared to both control plants, uninoculated (*P* < 0.01) and GFP-silenced (*P* < 0.05). The further reduction in the silenced plants result indicates that the residual editing extent of some sites in *orrm3* mutants is dependent on the presence of ORRM2.

### The N-terminal RRM domain of ORRM3 can rescue the editing defects in the *orrm3–1* mutant

**Figure 9.**
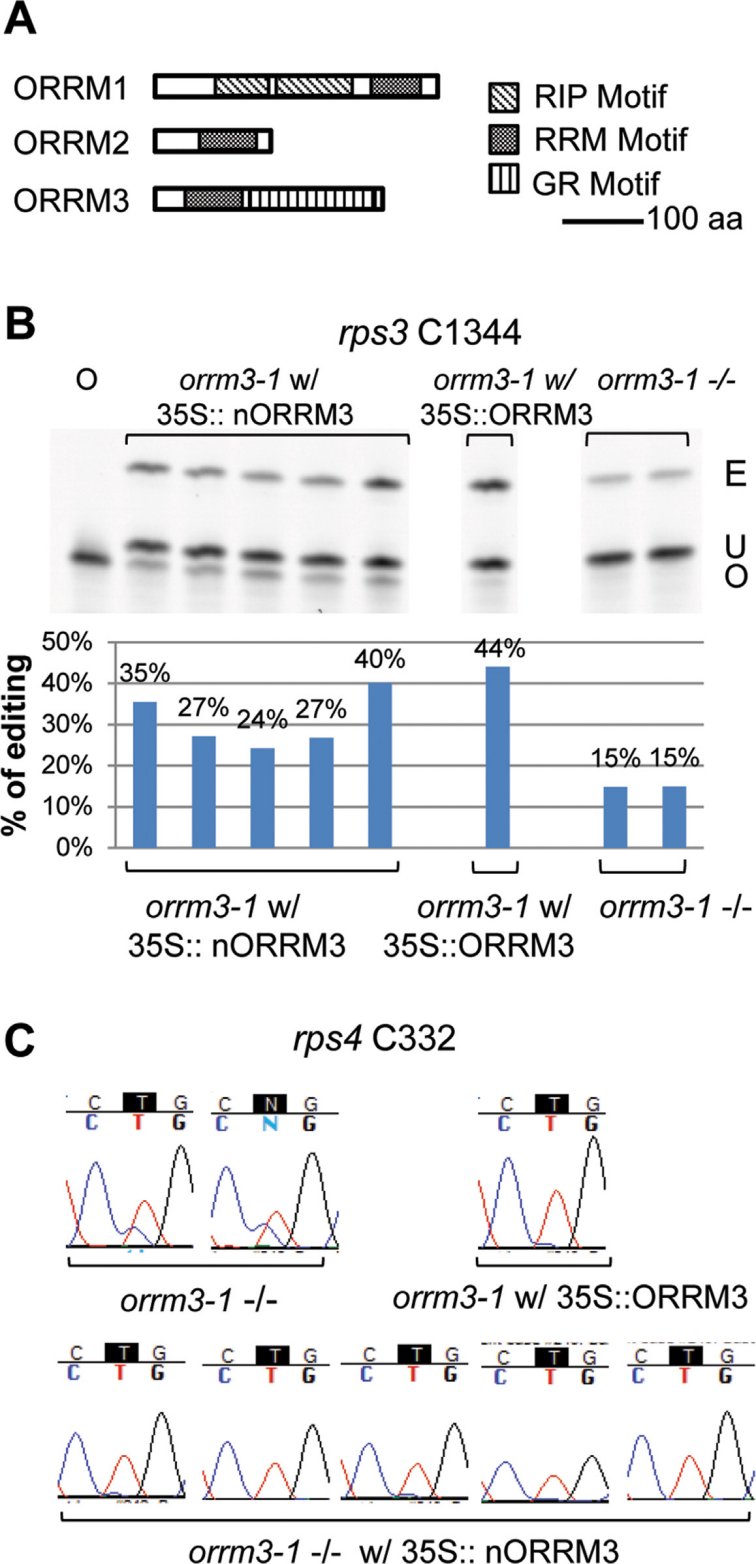
The N-terminal RRM domain of ORRM3 is able to rescue RNA editing activity in *orrm3* mutants. (**A**) The motif diagram of ORRM1, ORRM2 and ORRM3. (**B**) Editing at site *rps3* C1344 is restored in *orrm3* mutant plants by introducing the N-terminal RRM domain of ORRM3 as shown by PPE assay. E, edited band; U, unedited band; O, oligonucleotide. *orrm3–1* w/ 35S::nORRM3, *orrm3–1* mutant plants transformed with a construct expressing N-terminal RRM domain of ORRM3 driven by a 35S promoter; *orrm3–1* w/ 35S::ORRM3, *orrm3–1* mutant plants transformed with a construct expressing full-length ORRM3 driven by a 35S promoter; *orrm3–*1, homozygous *orrm3–1* mutant plant;. (**C**) Editing at site *rps4* C332 is restored in *orrm3* mutant plants by introducing the N-terminal RRM domain of ORRM3 as shown by a bulk sequencing assay. The editable C is shown in white letter in black background.

ORRM3 contains an RNA recognition motif at the N terminus and a Glycine rich (GR) domain at the C terminus (Figure 9A). To find out the role of the RRM domain of ORRM3 in RNA editing, we transformed homozygous *orrm3* mutant plants with a construct expressing the N-terminal RRM domain of ORRM3 under the control of a 35S promoter. All the transgenic plants exhibited a normal phenotype. The editing extents of several independent transgenic plants were analyzed by PPE and bulk sequencing assays. Mutants expressing the RRM domain of ORRM3 showed increased editing extent at the sites we monitored compared to *orrm3* mutants without the transgene (Figure 9B and C). At site *rps3* C1344, the editing extent in mutants expressing N-terminal ORRM3 varies from 24 to 40% (Figure 9B). As for the transgenic mutants expressing a full length *ORRM3* (Figure 5A), the variation of editing extents in different complemented plants is likely a reflection of differential expression of transgenes due to position effects. Considering that the editing extent of *orrm3* mutant plant is about 15% (Figure 9B), the expression of the N-terminal RRM domain of ORRM3 complements the defective editing in *orrm3* mutants. This conclusion is also supported by the fact that the editing at site *rps4* C332 is restored to ∼100% in mutants expressing either full-length ORRM3 or N-terminal ORRM3 (Figure 9C).

## DISCUSSION

Here we report the identification and characterization of ORRM2 and ORRM3, mitochondrial editing factors controlling the editing extent of about 6% of mitochondrial sites in Arabidopsis. This number certainly underestimates the realm of influence of these factors, as it was derived from silencing experiments where the expression of these genes was only knocked down and not eliminated. We performed T-DNA mutant analysis and stable complementation to support the results of the silencing experiments and confirm the function of ORRM3 as a mitochondrial editing factor. We could not perform similar mutant analysis and stable complementation of *orrm2* mutant plants because a homozygous T-DNA mutant in *ORRM2* coding sequence is not available in the mutant collections.

Unlike ORRM1, the founding member of the family of ORRM editing factors, in which a null mutation results in the abolition of editing at 12 plastid sites (20), the *orrm3* mutant did not show an absence of editing at any of the sites we assayed. It is likely that functional redundancy between *ORRM3* and other *ORRMs* prevents the complete elimination of editing at specific sites in the *orrm3* mutant. This hypothesis is supported by the large overlap of sites under the control of ORRM2 and ORRM3, as shown by the VIGS experiment. This result is not caused by an off-target effect of transient silencing. The silencing of *ORRM2* does not affect *ORRM3* expression and the silencing of *ORRM3* does not affect *ORRM2* expression.

In our Y2H assay, ORRM2 did not interact with the PPR protein MEF1, though both of them control the editing at site *nad7* C963. ORRM1 is able to interact with PPR proteins selectively in yeast and its RIP–RIP domain is required for the interaction (20). Furthermore, the selective binding of ORRM1 to PPRs in Y2H assays predicts the identity of the sites under its control. For example, ORRM1 was shown to interact with the PPR protein CRR28, which is required for the editing of *ndhB* C467 and *ndhD* C878 (32), two sites whose editing extent is severely reduced to more than 90% in the *orrm1* mutant (20). Conversely ORRM1 did not interact with the PPR protein RARE1, which is required for the editing of *accD* C794 (26), a site whose editing extent in the *orrm1* mutant is identical to the wild-type plant (20). The absence of a RIP domain in ORRM2 may explain why ORRM2 does not interact with MEF1 in yeast. ORRM3 interacts in yeast with RIP1, a major mitochondrial editing factor, further supporting the role of ORRM3 as an editing factor. Our data suggest that the selectivity of the sites under the control of ORRM3 might result from its interaction with RIP1, which itself interacts selectively with PPR recognition factors in a similar way as ORRM1 (T. Sun, unpublished results).

Like ORRM1, ORRM2 and ORRM3 carry one RRM domain. The RRM domain is one of the most abundant domains in eukaryotes and is involved in various aspects of RNA metabolism (33). Our previous study showed that the RRM domain in ORRM1 is able to provide the editing activity of ORRM1 in plastids (20). Now we demonstrate that the expression of the RRM domain in ORRM3 can restore the editing defect in *orrm3* mutants, indicating that RRM domain plays an important role in RNA editing in both plastids and mitochondria. The extreme structural versatility of the RRM domain explains the high variety of its interacting partners, not only RNA or DNA ligands but also proteins and consequently its involvement in a wide range of cellular processes (34). We have shown in previous work that ORRM1 has intrinsic specificity for sequences near at least some of its RNA targets (20). Therefore it is possible that some of the specificity of the sites under the control of ORRM2 and ORRM3 might be dependent on the ability of these factors to bind selectively near some of their RNA targets. Other RRM-containing proteins have been found to be RNA editing factors in systems other than plants. The mammalian ACF (apobec-1 complementation factor) is a RRM-containing protein which binds to *apo-B* mRNA and docks apobec-1 to deaminate the C target (35).

Another plant RRM-containing protein, CP31A, has a moderate effect on plastid editing and a major effect on RNA stability (36,37). CP31A was shown to be essential for resistance of chloroplast development to cold stress by guaranteeing transcript stability of numerous mRNAs at low temperature (37). We believe that the effect of ORRM2 and ORRM3 on mitochondrial editing is a more direct one, because editing defects in silenced or mutant plants are site specific and not transcript specific.

ORRM3, which contains a GR domain at its C-terminus, was previously described as GR-RBP3 (glycine-rich RNA-binding protein 3) or At-mRBP2b (mitochondrial RNA-binding protein 2b) (30,38). A potato homolog to At-mRBP2b was isolated and identified in purified potato mitochondria by affinity chromatography to ssDNA. ORRM3/At mRBP2b has a much higher affinity to poly(U) than to other three homoribopolymers or to DNA (30). ORRM3 was also detected in a yeast-three hybrid screening assay based on its affinity to the 3′ UTR of *StBEL5*, a key element in regulating RNA metabolism (39). This further supports the possibility that ORRM3 is able to bind RNA within the editing machinery. Little was previously known about ORRM2, except that it exhibits affinity to ssDNA and was first identified based on its homology to mRBP proteins (30).

Plant glycine-rich proteins (GRPs) are characterized by the presence of a GR domain arranged in (Gly)_n_-X repeats, usually with a specific expression pattern modulated by several biotic and abiotic factors (40). Some GRPs are accompanied by other domains such as cysteine-rich domains, cold-shock domains, CCHC zinc-finger domains, etc. Among the GR family of proteins, ORRM3 is classified as Class IVa based on its N-terminal RRM domain (40). RNA-binding GRPs or GR-RBPs have been shown to be involved in the environmental stress responses of plants, especially cold and salt stress responses (30,41,42). GR-RBP2/GRP2 was found to be involved in cold and salt stress responses, whereas expression of GR-RBP4/GRP4 was modulated by cold and osmotic stress (43,44). However, the molecular mechanism of action of these proteins is still unknown. Thus this study implicates a new molecular function for GR-RBPs.

The identification of members of the ORRM clade as mitochondrial RNA editing factors further expands our knowledge of the composition of the editosome. It also supports the monophyletic origin of editing in both organelles. So far, every family of plant editing factors, PPR proteins, RIP/MORF proteins and now ORRM proteins, has members involved in either mitochondrial or plastid editing, and sometimes in both, like RIP1. Another outcome of this study is the confirmation that plant editosomes are heterogeneous in their composition. The complement of proteins required for editing of a particular C target within mitochondria evidently differs not merely by the PPR recognition factor, but also with regard to members of two additional protein families.

## SUPPLEMENTARY DATA

Supplementary Data are available at NAR Online.

SUPPLEMENTARY DATA
