# Supplementary material for: Two RNA recognition motif-containing proteins are plant mitochondrial editing factors
Source: Nucleic Acids Res. 2015 Mar 23;43(7):3814–25. doi: 10.1093/nar/gkv245 (PMC4402546; doi:10.1093/nar/gkv245)
Supplement: SUPPLEMENTARY DATA [file supp_43_7_3814__index.html]

Two RNA recognition motif-containing proteins are plant mitochondrial editing factors — Two RNA recognition motif-containing proteins are plant mitochondrial editing factors — SUPPLEMENTARY DATA 

# Two RNA recognition motif-containing proteins are plant mitochondrial editing factors

## SUPPLEMENTARY DATA

**Files in this Data Supplement:**

- SUPPLEMENTARY DATA
- SUPPLEMENTARY DATA
- SUPPLEMENTARY DATA
