# Supplementary material for: Two RNA recognition motif-containing proteins are plant mitochondrial editing factors
Source: Nucleic Acids Res. 2015 Mar 23;43(7):3814–25. doi: 10.1093/nar/gkv245 (PMC4402546; doi:10.1093/nar/gkv245)
Supplement: SUPPLEMENTARY DATA [file supp_gkv245_nar-00333-y-2015-File012.pdf]

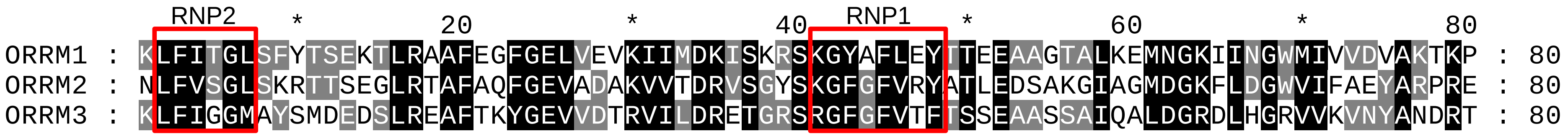

Supplementary Figure S1. Alignment of the RNA Recognition Motifs (RRMs) found in ORRM1, ORRM2, and ORRM3. The RRM domain is ~80-aa long and contains two short consensus sequences, RNP1 (octamer) and RNP2 (hexamer), which are characteristic of RRMs. Alignment was performed by T-Coffee version_9.03, and displayed using GeneDoc with the conserved residue shading mode and similarity groups enabled.

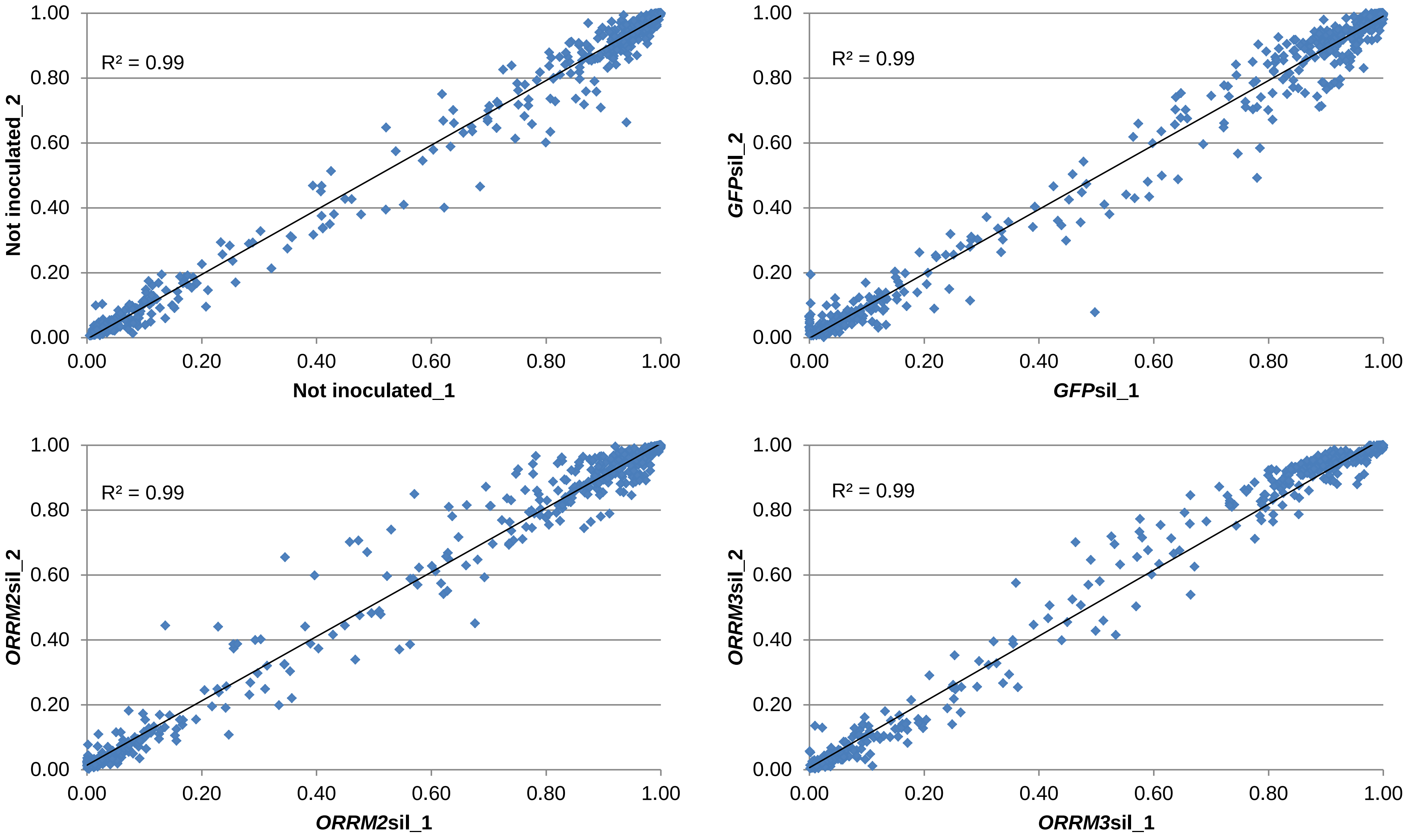

Supplementary Figure S2. Biological replicates exhibit a high correlation of editing extent measured by STS-PCRseq. Each graph represents a pairwise comparison of editing extent that was measured on two libraries obtained from cDNAs of two plants grown in the same conditions and harvested at the same time. Not inoculated, plants not inoculated with Agrobacteria. *GFP*-sil, plants inoculated with Agrobacteria harboring a *GFP* silencing construct. *ORRM2*-sil, plants inoculated with Agrobacteria harboring a *GFP* and *ORRM2* co-silencing construct. *ORRM3*-sil, plants inoculated with Agrobacteria harboring a *GFP* and *ORRM3* co-silencing construct. The correlation was calculated with 656 points: 618 mitochondrial sites and 38 plastid sites.

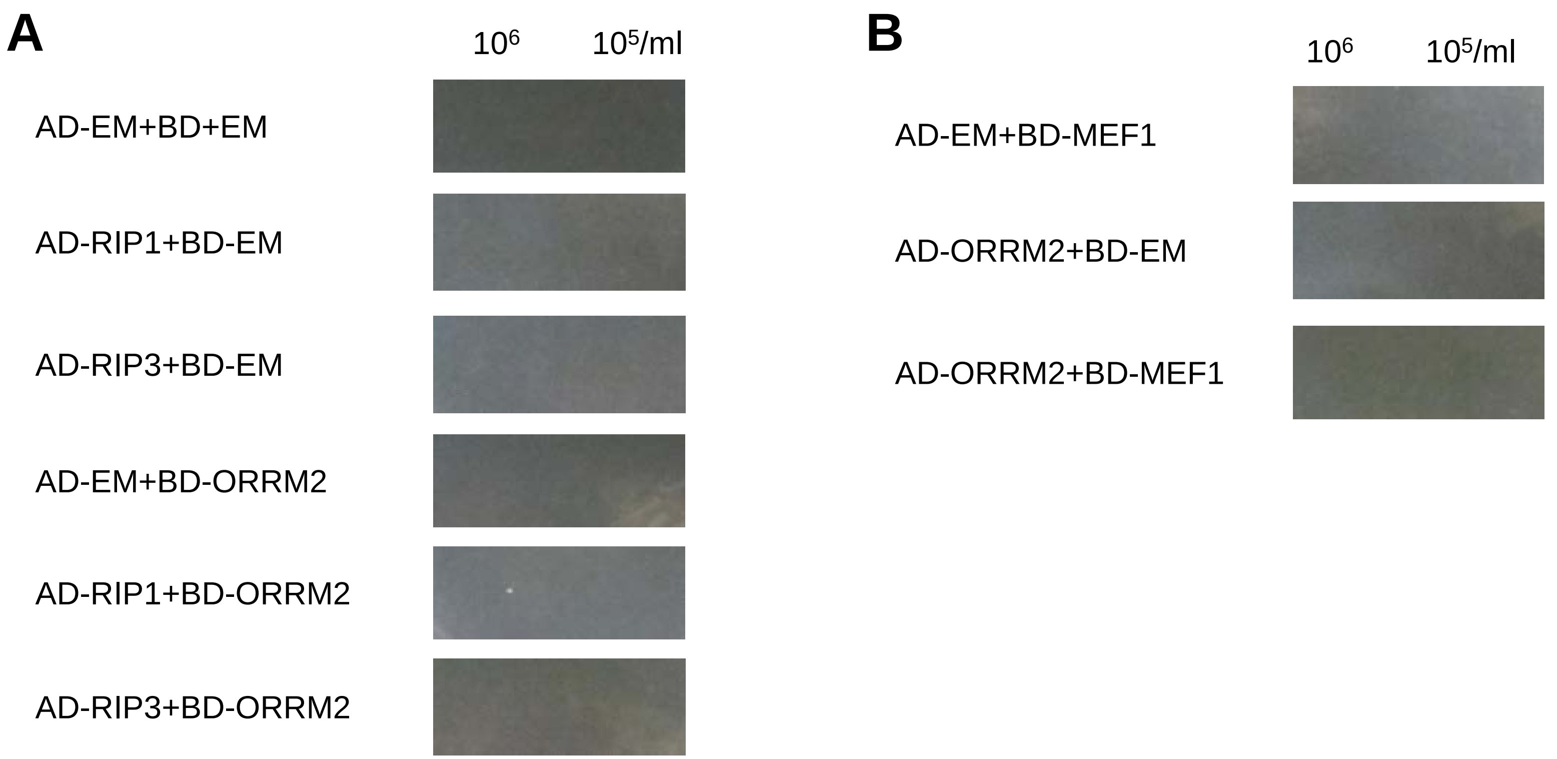

Supplementary Figure S3. ORRM2 does not interact with RIP1, RIP3 or MEF1. (**A**) ORRM2 does not interact with RIP1 or RIP3. (**B**) ORRM2 does not interact with MEF1 though editing at site *nad7* C963 is reduced in both *ORRM2*-silenced plants and MEF1 mutant plants. EM, yeasts transformed with a vector carrying an empty GW cassette as a negative control.

Supplementary Table S1. Primers used in this study

ORRM3-vigs-F	ACACTTATGGGGAGGGTTC
ORRM3-vigs-R	CCTATTGGTCCATCTTCCATCTT
ORRM2-vigs-F	ATATGCCACTTTAGAAGATTCTGCC
ORRM2-vigs-R	AATAACACGAGACGAAGCACA
SALK_038244-LP	TGCGTGGATGGAATTCTTAAG
SALK_038244-RP	TTGGATCGTGAAACTGGTAGG
ORRM3-F	ATGGCTTTTTTGAGTAAATTCGGGAAC
ORRM3-R	TCAAGCTCTTTTGGCAACAT
ORRM3 w/o stop-R	AGCTCTTTTGGCAACATCTT
ORRM3-109F	ATGTCATCTTCTAAGCTCTTTATTGG
ORRM2-F	ATGGCGATGGCTATGAGACT
ORRM2-R	CTAGCGGTTACCATAGTAT
ORRM2 w/o stop-R	GCGGTTACCATAGTATGGAGGA
ORRM2-103F	TTCTCTTTTTTGTCTCCACAAGC
HA-R	GCACTGCAGTTATCAAGCGTAGTCAGGTACGTC
PPE-rps4-77R	GTCAGTTCTCTGTTCCAAACATTTCC
PPE-orf240A-199	GACTTGGAAAAAATCGTAGTTCAGATTCAAG
PPE-rps3-1344R	AAGAGATGTCTTGAGCAATCAGAGAAGC
ORRM3-360R	CTATGTTCTATCATTTGCATAGT
PPE-nad6ldC-73F	AAATGAAATAGGAACAACCGCGCTGG
PBI121-F	GGGATGACGCACAATCCCACTATCC
qRT-ORRM2-F	TTTTTCCCGAAGAAGAATCAATACGACTC
qRT-ORRM2-R	AAACCAACCGGAGCTGAAGCTACTTC
qRT-ORRM3-F	ATGTTGCCAAAAGAGCTTGAAGGAACTA
qRT-ORRM3-R	AAGATAGCAAAAGCAGATTGTTTCTTGA

Supplementary Table S2 . Sites showing a significant reduction of editing extent in *ORRM2* -silenced sites

#Gene	Position ATG	Editing extent						ΔrORRM2sil_1	P ($\chi^2_{ORRM2\ sil\_1}$)	ΔrORRM2sil_2	P ($\chi^2_{ORRM2\ sil\_2}$)
		ORRM2 sil_1	*ORRM2* sil_2	Not inoculated	GFPsil	(Not inoc-ORRM2sil_1)	(Not inoc-ORRM2sil_2)				
nad7	795	0.26	0.39	0.84	0.76	0.58	0.45	0.69	0.E+00	0.54	0.E+00
nad6leader	-73	0.40	0.60	0.87	0.85	0.47	0.27	0.54	0.E+00	0.31	0.E+00
nad7	963	0.57	0.85	0.99	0.99	0.42	0.14	0.43	0.E+00	0.14	2.E-157
ccmB	80	0.47	0.71	0.86	0.78	0.39	0.15	0.45	5.E-303	0.18	3.E-67
nad5intron	141966	0.07	0.18	0.40	0.36	0.32	0.21	0.82	0.E+00	0.54	3.E-216
rpl5	59	0.46	0.70	0.78	0.73	0.32	0.07	0.41	3.E-250	0.10	5.E-21
ccmFc	406	0.53	0.74	0.84	0.82	0.31	0.11	0.37	3.E-115	0.12	1.E-28
rps3	1344	0.14	0.17	0.45	0.42	0.31	0.29	0.68	3.E-231	0.63	1.E-131
nad4	1101	0.57	0.59	0.86	0.90	0.30	0.28	0.34	4.E-164	0.32	2.E-125
ccmC	618	0.71	0.70	0.93	0.89	0.23	0.24	0.24	0.E+00	0.25	0.E+00
orf114	327	0.45	0.44	0.65	0.66	0.20	0.21	0.31	3.E-131	0.32	1.E-165
nad7	213	0.78	0.86	0.98	0.98	0.19	0.12	0.20	3.E-142	0.12	7.E-87
nad72intron	134309	0.76	0.86	0.95	0.94	0.19	0.09	0.20	0.E+00	0.10	2.E-229
ccmC	463	0.79	0.83	0.95	0.94	0.16	0.12	0.17	6.E-80	0.13	3.E-54
nad3	26	0.78	0.79	0.93	0.93	0.15	0.14	0.16	5.E-135	0.15	3.E-133
mttb	59	0.77	0.79	0.92	0.92	0.15	0.13	0.16	9.E-71	0.14	8.E-64
ccmFc	1215	0.52	0.60	0.67	0.70	0.15	0.07	0.22	2.E-46	0.11	2.E-21
rps14	99	0.04	0.07	0.17	0.16	0.13	0.10	0.78	7.E-138	0.58	9.E-98
ccmC	184	0.76	0.71	0.87	0.84	0.11	0.16	0.13	8.E-97	0.18	7.E-199
ccmB	406	0.24	0.26	0.34	0.37	0.10	0.09	0.29	8.E-15	0.25	6.E-13
nad4	1131	0.02	0.04	0.12	0.10	0.09	0.07	0.79	1.E-37	0.62	1.E-17
ccmC	270	0.06	0.08	0.15	0.13	0.09	0.08	0.61	3.E-52	0.50	1.E-39
nad4	1194	0.05	0.06	0.14	0.11	0.09	0.08	0.61	1.E-27	0.60	4.E-20
rpl16	72	0.00	0.02	0.07	0.10	0.07	0.05	0.96	2.E-47	0.71	1.E-51
rps7leader	-217	0.11	0.13	0.18	0.25	0.07	0.05	0.40	2.E-19	0.29	2.E-13
cob	114	0.01	0.02	0.07	0.07	0.06	0.05	0.83	1.E-40	0.69	3.E-46
ccmFc	315	0.00	0.01	0.05	0.03	0.05	0.04	0.96	1.E-16	0.75	7.E-22
rps4	711	0.03	0.03	0.07	0.08	0.04	0.04	0.60	4.E-15	0.54	1.E-15
nad72intron	134282	0.01	0.02	0.05	0.07	0.04	0.03	0.77	6.E-74	0.61	4.E-45
rps12	328	0.00	0.01	0.04	0.04	0.04	0.03	0.97	6.E-70	0.70	3.E-47
nad5	1494	0.01	0.01	0.04	0.04	0.03	0.02	0.83	6.E-12	0.64	8.E-11
rps3	270	0.01	0.01	0.03	0.05	0.03	0.02	0.78	3.E-21	0.61	4.E-08
nad9	517	0.03	0.02	0.05	0.05	0.02	0.03	0.45	5.E-12	0.53	3.E-22
nad5	1933	0.01	0.01	0.03	0.02	0.02	0.02	0.66	3.E-09	0.58	1.E-10
nad2	336	0.01	0.01	0.03	0.06	0.02	0.02	0.60	1.E-06	0.59	1.E-11

Sites have been sorted according to a decreasing editing extent (Not inoc-*ORRM2* sil_1). Δ*ORRM2* sil_n: (Not inoc-*ORRM2* sil_n)/Not inoc. Sites which are also reduced in *ORRM3* -silenced tissues are shown in a blue background.

Supplementary Table S3 . Sites showing a significant reduction of editing extent in *ORRM3* -silenced sites

#Gene	Position ATG	Editing extent						ΔORRM3sil_1	P ($\chi^2_{ORRM3\ sil\_1}$)	Δ*ORRM3* sil_2	P ($\chi^2_{ORRM3\ sil\_2}$)
		ORRM3 **sil_1**	***ORRM3*** **sil_2**	**Not inoculated**	**GFPsil**	**(Not inoc-*ORRM3sil* _1)**	**(Not inoc-*ORRM3* sil_2)**				
ccmC	618	0.46	0.70	0.93	0.89	0.47	0.23	0.50	0.E+00	0.25	1.E-283
rps3	1344	0.08	0.13	0.45	0.42	0.37	0.33	0.83	2.E-195	0.72	7.E-139
nad6leade	-73	0.54	0.63	0.87	0.85	0.33	0.24	0.38	0.E+00	0.27	4.E-259
nad5intror	141966	0.10	0.16	0.40	0.36	0.30	0.23	0.76	0.E+00	0.59	8.E-152
orf114	327	0.36	0.58	0.65	0.66	0.29	0.07	0.45	0.E+00	0.11	2.E-22
ccmC	463	0.66	0.85	0.95	0.94	0.29	0.10	0.30	5.E-182	0.11	3.E-37
orf240A	199	0.21	0.29	0.43	0.48	0.22	0.14	0.51	9.E-121	0.32	3.E-38
ccmC	252	0.25	0.35	0.45	0.41	0.20	0.10	0.44	7.E-97	0.21	2.E-19
rps4	77	0.59	0.68	0.78	0.79	0.19	0.10	0.24	2.E-188	0.13	2.E-59
ccmC	381	0.18	0.21	0.32	0.29	0.14	0.11	0.45	3.E-46	0.34	6.E-21
ccmC	270	0.04	0.05	0.15	0.13	0.12	0.10	0.77	9.E-85	0.67	9.E-47
nad5	609	0.79	0.77	0.87	0.90	0.08	0.10	0.10	1.E-16	0.12	3.E-22
nad4	1131	0.04	0.05	0.12	0.10	0.08	0.07	0.69	5.E-30	0.60	3.E-24
nad7	1050	0.09	0.14	0.17	0.18	0.08	0.03	0.45	5.E-40	0.19	6.E-07
nad4	1194	0.06	0.06	0.14	0.11	0.08	0.08	0.54	1.E-22	0.56	3.E-26
nad4	636	0.03	0.04	0.10	0.10	0.07	0.07	0.71	5.E-32	0.64	1.E-29
nad5	1665	0.05	0.05	0.12	0.11	0.07	0.07	0.58	5.E-28	0.57	5.E-24
atp1	1110	0.02	0.02	0.09	0.06	0.06	0.06	0.74	4.E-15	0.71	6.E-29
ccmB	75	0.09	0.06	0.15	0.13	0.06	0.08	0.39	9.E-17	0.57	5.E-39
nad1	40	0.13	0.10	0.19	0.18	0.06	0.08	0.30	2.E-18	0.44	4.E-33
nad7	789	0.19	0.14	0.25	0.27	0.06	0.10	0.23	1.E-07	0.41	1.E-21
mttb	422	0.02	0.03	0.07	0.05	0.05	0.04	0.76	3.E-30	0.64	3.E-15
nad5	1929	0.03	0.04	0.08	0.08	0.05	0.05	0.59	6.E-33	0.57	2.E-26
cob	114	0.02	0.03	0.07	0.07	0.05	0.04	0.71	6.E-35	0.62	5.E-38
cox2	250	0.04	0.03	0.08	0.09	0.04	0.05	0.55	1.E-16	0.61	1.E-25
ccmB	78	0.01	0.03	0.05	0.05	0.04	0.02	0.72	2.E-23	0.44	3.E-08
mttb	673	0.01	0.02	0.05	0.04	0.03	0.03	0.69	7.E-15	0.58	5.E-08
mttb	82	0.00	0.00	0.03	0.02	0.03	0.03	0.94	6.E-72	0.87	2.E-37
rps12	328	0.01	0.01	0.04	0.04	0.03	0.03	0.75	2.E-53	0.85	4.E-58
nad9	517	0.03	0.02	0.05	0.05	0.02	0.03	0.46	1.E-15	0.67	7.E-36
nad4	261	0.01	0.01	0.03	0.04	0.02	0.02	0.74	8.E-13	0.51	1.E-06
nad5	1933	0.01	0.01	0.03	0.02	0.02	0.02	0.61	6.E-12	0.67	5.E-13

Sites have been sorted according to a decreasing editing extent (Not inoc-*ORRM3* sil_1). Δ*ORRM3* sil_n: (Not inoc-*ORRM3* sil_n)/Not inoc. Sites which are also reduced in *ORRM2* -silenced tissues are shown in a blue background.

Supplementary Table S4 . Plastid editing extent in *ORRM2* -silenced sites

#Gene	Position ATG	Editing extent						Δ*ORRM2* sil_1	P ($\chi^2_{ORRM2\ sil\_1}$)	Δ*ORRM2* sil_2	P ($\chi^2_{ORRM2\ sil\_2}$)
		ORRM2 sil_1	*ORRM2* sil_2	Not inoculated	GFPsil	(Not inoc-*ORRM2sil* _1)	(Not inoc-*ORRM2* sil_2)				
accD	794	1.00	1.00	0.99	1.00	-0.01	-0.01	-0.01	8.E-02	-0.01	9.E-04
accD	1568	0.84	0.85	0.80	0.82	-0.05	-0.05	-0.06	2.E-03	-0.07	4.E-07
atpF	92	0.99	0.99	0.99	0.99	0.00	0.00	0.00	3.E-01	0.00	4.E-03
clpP	559	0.97	0.99	0.97	0.97	-0.01	-0.02	-0.01	7.E-02	-0.02	1.E-13
matK	640	0.95	0.95	0.92	0.94	-0.03	-0.03	-0.03	7.E-05	-0.03	6.E-05
ndhB	149	1.00	0.99	1.00	0.99	0.00	0.00	0.00	2.E-01	0.00	3.E-02
ndhB	467	0.98	0.99	0.99	0.99	0.01	0.00	0.01	1.E-02	0.00	2.E-01
ndhB	586	1.00	1.00	0.99	0.99	0.00	0.00	0.00	4.E-01	0.00	3.E-01
ndhB	726	0.10	0.09	0.09	0.08	-0.01	0.00	-0.06	6.E-01	-0.01	9.E-01
ndhB	746	1.00	1.00	1.00	1.00	0.00	0.00	0.00	4.E-01	0.00	4.E-01
ndhB	830	1.00	1.00	0.99	0.99	0.00	0.00	0.00	6.E-02	0.00	7.E-01
ndhB	836	0.99	0.99	0.99	0.99	0.00	0.00	0.00	9.E-01	0.00	8.E-01
ndhB	872	0.99	0.99	0.99	0.99	0.00	0.00	0.00	7.E-01	0.00	9.E-01
ndhB	1255	1.00	1.00	1.00	1.00	0.00	0.00	0.00	7.E-01	0.00	4.E-01
ndhB	1481	1.00	1.00	1.00	1.00	0.00	0.00	0.00	8.E-01	0.00	7.E-01
ndhD	2	0.62	0.54	0.48	0.48	-0.15	-0.07	-0.31	1.E-33	-0.14	5.E-09
ndhD	383	0.99	0.99	0.99	0.99	0.00	0.00	0.00	1.E+00	0.00	9.E-01
ndhD	674	0.97	0.94	0.95	0.92	-0.02	0.01	-0.02	2.E-05	0.01	2.E-02
ndhD	878	0.94	0.88	0.89	0.82	-0.05	0.01	-0.05	3.E-11	0.01	1.E-01
ndhD	887	0.95	0.90	0.90	0.85	-0.05	0.00	-0.05	1.E-13	0.00	8.E-01
ndhF	290	1.00	1.00	1.00	0.99	0.00	0.00	0.00	3.E-01	0.00	2.E-01
ndhF	932	0.03	0.02	0.03	0.03	0.00	0.01	0.04	8.E-01	0.27	6.E-02
ndhG	50	0.89	0.90	0.88	0.88	-0.01	-0.02	-0.01	2.E-01	-0.03	8.E-04
petL	5	0.99	0.97	0.98	0.96	0.00	0.01	0.00	8.E-01	0.01	3.E-01
psbE	214	1.00	1.00	1.00	1.00	0.00	0.00	0.00	1.E-01	0.00	1.E-01
psbF	77	1.00	0.99	0.99	0.99	-0.01	0.00	-0.01	3.E-01	0.00	9.E-01
psbZ	50	0.97	0.98	0.97	0.98	0.00	-0.01	0.00	3.E-01	-0.01	2.E-02
rpl23	89	0.90	0.95	0.91	0.92	0.01	-0.03	0.01	3.E-02	-0.04	4.E-16
rpoA	200	0.90	0.93	0.87	0.94	-0.03	-0.06	-0.03	3.E-03	-0.07	5.E-16
rpoB	338	0.94	0.92	0.93	0.93	-0.02	0.01	-0.02	5.E-04	0.01	9.E-03
rpoB	551	0.95	0.93	0.94	0.94	-0.01	0.01	-0.01	2.E-02	0.01	4.E-02
rpoB	2432	0.94	0.97	0.94	0.98	0.00	-0.03	0.00	6.E-01	-0.03	1.E-31
rpoC1	488	0.35	0.30	0.29	0.32	-0.06	-0.01	-0.22	2.E-05	-0.05	3.E-01
rps14	80	0.99	0.99	0.99	0.98	0.00	0.00	0.00	9.E-01	0.00	7.E-01
rps14	149	0.96	0.96	0.94	0.94	-0.01	-0.01	-0.02	2.E-05	-0.02	9.E-05

Supplementary Table S5 . Plastid editing extent in *ORRM3* -silenced sites

#Gene	Position ATG	Editing extent						ΔORRM3sil_1	P ($\chi^2_{ORRM3\ sil\_1}$)	Δ*ORRM3* sil_2	P ($\chi^2_{ORRM3\ sil\_2}$)
		***ORRM3* sil_1**	***ORRM3* sil_2**	**Not inoculated**	**GFPsil**	**(Not inoc-*ORRM3sil* _1)**	**(Not inoc-*ORRM3* sil_2)**				
accD	794	1.00	1.00	0.99	1.00	-0.01	-0.01	-0.01	2.E-03	-0.01	3.E-02
accD	1568	0.83	0.85	0.80	0.82	-0.03	-0.05	-0.04	9.E-03	-0.07	6.E-06
atpF	92	0.99	0.99	0.99	0.99	0.00	-0.01	0.00	4.E-04	-0.01	6.E-04
clpP	559	0.98	0.99	0.97	0.97	-0.01	-0.02	-0.01	2.E-05	-0.02	2.E-15
matK	640	0.90	0.92	0.92	0.94	0.02	0.00	0.02	2.E-02	0.00	1.E+00
ndhB	149	0.99	1.00	1.00	0.99	0.00	0.00	0.00	4.E-04	0.00	3.E-01
ndhB	467	0.98	0.99	0.99	0.99	0.01	0.00	0.01	4.E-03	0.00	9.E-01
ndhB	586	0.99	0.99	0.99	0.99	0.00	0.00	0.00	3.E-02	0.00	8.E-01
ndhB	726	0.09	0.08	0.09	0.08	0.00	0.01	-0.01	9.E-01	0.10	2.E-01
ndhB	746	1.00	1.00	1.00	1.00	0.00	0.00	0.00	4.E-01	0.00	7.E-01
ndhB	830	0.99	0.99	0.99	0.99	0.00	0.00	0.00	7.E-01	0.00	1.E+00
ndhB	836	0.98	0.99	0.99	0.99	0.01	0.00	0.01	1.E-08	0.00	4.E-01
ndhB	872	0.98	0.99	0.99	0.99	0.01	0.00	0.01	4.E-05	0.00	3.E-01
ndhB	1255	1.00	1.00	1.00	1.00	0.00	0.00	0.00	4.E-01	0.00	1.E-01
ndhB	1481	0.99	1.00	1.00	1.00	0.00	0.00	0.00	5.E-01	0.00	5.E-01
ndhD	2	0.51	0.58	0.48	0.48	-0.03	-0.10	-0.06	2.E-03	-0.22	6.E-26
ndhD	383	0.99	0.99	0.99	0.99	0.00	0.00	0.00	3.E-01	0.00	3.E-01
ndhD	674	0.89	0.96	0.95	0.92	0.06	-0.01	0.06	4.E-28	-0.01	2.E-01
ndhD	878	0.80	0.91	0.89	0.82	0.09	-0.01	0.11	7.E-39	-0.01	3.E-02
ndhD	887	0.83	0.92	0.90	0.85	0.07	-0.02	0.08	5.E-26	-0.02	4.E-03
ndhF	290	0.99	1.00	1.00	0.99	0.01	0.00	0.01	5.E-02	0.00	2.E-01
ndhF	932	0.02	0.01	0.03	0.03	0.01	0.02	0.28	9.E-02	0.56	6.E-04
ndhG	50	0.88	0.91	0.88	0.88	0.00	-0.03	0.00	8.E-01	-0.03	3.E-03
petL	5	0.96	0.95	0.98	0.96	0.02	0.03	0.02	1.E-01	0.03	3.E-02
psbE	214	1.00	1.00	1.00	1.00	0.00	0.00	0.00	4.E-01	0.00	4.E-01
psbF	77	0.99	0.99	0.99	0.99	0.01	0.00	0.01	4.E-01	0.00	7.E-01
psbZ	50	0.97	0.98	0.97	0.98	0.01	0.00	0.01	9.E-02	0.00	5.E-01
rpl23	89	0.91	0.91	0.91	0.92	0.01	0.01	0.01	5.E-02	0.01	2.E-01
rpoA	200	0.94	0.94	0.87	0.94	-0.07	-0.07	-0.08	4.E-19	-0.08	4.E-17
rpoB	338	0.95	0.88	0.93	0.93	-0.03	0.05	-0.03	5.E-13	0.05	2.E-17
rpoB	551	0.96	0.90	0.94	0.94	-0.02	0.04	-0.02	3.E-06	0.05	5.E-15
rpoB	2432	0.97	0.91	0.94	0.98	-0.02	0.03	-0.03	7.E-20	0.03	2.E-28
rpoC1	488	0.33	0.33	0.29	0.32	-0.04	-0.04	-0.13	2.E-03	-0.13	4.E-03
rps14	80	0.97	0.98	0.99	0.98	0.02	0.00	0.02	6.E-11	0.00	2.E-01
rps14	149	0.95	0.95	0.94	0.94	-0.01	0.00	-0.01	2.E-03	0.00	2.E-01

In blue shading is indicated the only site that shows a significant reduction of editing extent (P<2.6 e-5 and Δ ≥ 0.1) only for one of the *ORRM3* -silenced individual (*ORRM3* sil_1) .

Supplementary Table 6. Effect of *RIP1* or *RIP3* mutation on sites showing a significant reduction of editing extent in *ORRM3*-silenced sites

#Gene	Position ATG	Editing extent				Δ*ORRM3* sil_1	Δ*RIP1*	P (χ^2_{RIP1})	Δ*RIP3-1*	P (χ^2_{RIP3-1})	Δ*RIP3-2*	P (χ^2_{RIP3-2})
		ORRM3 sil_1	Not inoculated	GFPsil	(Not inoculated-*ORRM3sil*_1)							
atp1	1110	0.02	0.09	0.06	0.06	0.74	0.96	2.E-105	0.98	0.E+00	0.94	4.E-94
ccmB	75	0.09	0.15	0.13	0.06	0.39	0.95	4.E-120	0.25	2.E-06	0.2	5.E-08
ccmB	78	0.01	0.05	0.05	0.04	0.72	0.89	2.E-40	0.14	2.E-01	0.18	3.E-02
ccmC	252	0.25	0.45	0.41	0.20	0.44	0.71	0.E+00	0.27	4.E-146	0.06	9.E-03
ccmC	270	0.04	0.15	0.13	0.12	0.77	0.76	4.E-125	0.69	0.E+00	0.46	2.E-35
ccmC	381	0.18	0.32	0.29	0.14	0.45	0.50	4.E-11	0.25	2.E-55	0.22	1.E-11
ccmC	463	0.66	0.95	0.94	0.29	0.30	0.00	1.E-01	-0.01	5.E-02	0.02	1.E-02
ccmC	618	0.46	0.93	0.89	0.47	0.50	0.94	0.E+00	-0.02	6.E-02	0	9.E-01
cob	114	0.02	0.07	0.07	0.05	0.71	0.75	3.E-39	-2.09	4.E-81	-2.37	4.E-35
cox2	250	0.04	0.08	0.09	0.04	0.55	0.82	7.E-48	-0.34	2.E-12	-0.36	2.E-08
mttb	82	0.00	0.03	0.02	0.03	0.94	0.64	1.E-04	0.29	4.E-06	0.62	7.E-19
mttb	422	0.02	0.07	0.05	0.05	0.76	0.94	7.E-41	0.93	3.E-157	0.95	4.E-49
mttb	673	0.01	0.05	0.04	0.03	0.69	0.73	5.E-07	0.80	2.E-32	0.94	1.E-39
nad1	40	0.13	0.19	0.18	0.06	0.30	0.56	8.E-13	0.19	6.E-25	-0.17	4.E-04
nad4	261	0.01	0.03	0.04	0.02	0.74	0.43	2.E-01	0.90	4.E-171	0.95	2.E-135
nad4	636	0.03	0.10	0.10	0.07	0.71	0.82	1.E-33	-0.31	4.E-13	-0.65	2.E-08
nad4	1131	0.04	0.12	0.10	0.08	0.69	0.82	9.E-35	-0.07	5.E-02	-0.28	3.E-09
nad4	1194	0.06	0.14	0.11	0.08	0.54	0.85	4.E-75	0.88	4.E-260	0.84	1.E-58
nad5	609	0.79	0.87	0.90	0.08	0.10	0.88	2.E-180	0.62	0.E+00	0.56	0.E+00
nad5	1665	0.05	0.12	0.11	0.07	0.58	0.35	6.E-06	0.16	1.E-11	0.18	4.E-04
nad5	1929	0.03	0.08	0.08	0.05	0.59	0.75	2.E-10	-0.12	2.E-05	0.36	1.E-07
nad5	1933	0.01	0.03	0.02	0.02	0.61	0.78	6.E-07	-0.10	7.E-02	0.53	8.E-11
nad5intron	141966	0.10	0.40	0.36	0.30	0.76	0.91	1.E-250	-0.12	5.E-48	0.03	4.E-01
nad6leader	-73	0.54	0.87	0.85	0.33	0.38	0.84	0.E+00	0.04	1.E-132	0.02	2.E-02
nad7	789	0.19	0.25	0.27	0.06	0.23	0.07	3.E-02	-0.13	9.E-08	0.15	4.E-08
nad7	1050	0.09	0.17	0.18	0.08	0.45	-0.66	2.E-37	-0.10	1.E-05	-0.07	1.E-02
nad9	517	0.03	0.05	0.05	0.02	0.46	0.95	0.E+00	0.16	7.E-09	0.22	4.E-08
orf114	327	0.36	0.65	0.66	0.29	0.45	0.72	2.E-262	0.85	0.E+00	0.84	0.E+00
orf240A	199	0.21	0.43	0.48	0.22	0.51	-0.85	9.E-268	-0.39	8.E-241	-0.09	1.E-02
rps12	328	0.01	0.04	0.04	0.03	0.75	0.93	6.E-18	0.00	1.E+00	-0.37	4.E-05
rps3	1344	0.08	0.45	0.42	0.37	0.83	0.98	0.E+00	0.98	0.E+00	0.98	0.E+00
rps4	77	0.59	0.78	0.79	0.19	0.24	0.97	0.E+00	0.83	0.E+00	0.8	0.E+00

Sites showing a significant reduction of editing extent in *rip1* and *rip3* mutants are shown in a blue background (data from Bentolila et al., 2013). Two independent *rip3* insertional mutants were assessed; only sites showing reduction in both mutants are highlighted.
