# Supplementary material for: Two RNA recognition motif-containing proteins are plant mitochondrial editing factors
Source: Nucleic Acids Res. 2015 Mar 23;43(7):3814–25. doi: 10.1093/nar/gkv245 (PMC4402546; doi:10.1093/nar/gkv245)
Supplement: SUPPLEMENTARY DATA [file supp_gkv245_nar-00333-y-2015-File011.docx]

**SUPPLEMENTARY DATA**

**Supplementary Figure S1.** Alignment of the RNA Recognition Motifs (RRMs) found in ORRM1, ORRM2, and ORRM3. The RRM domain is ~80-aa long and contains two short consensus sequences, RNP1 (octamer) and RNP2 (hexamer), which are characteristic of RRMs. Alignment was performed by T-Coffee version_9.03, and displayed using GeneDoc with the conserved residue shading mode and similarity groups enabled.

**Supplementary Figure S2.** Biological replicates exhibit a high correlation of editing extent measured by STS-PCRseq. Each graph represents a pairwise comparison of editing extent that was measured on two libraries obtained from cDNAs of two plants grown in the same conditions and harvested at the same time. Not inoculated, plants not inoculated with Agrobacteria. *GFP*-sil, plants inoculated with Agrobacteria harboring a *GFP* silencing construct. *ORRM2*-sil, plants inoculated with Agrobacteria harboring a *GFP* and *ORRM2* co-silencing construct. *ORRM3*-sil, plants inoculated with Agrobacteria harboring a *GFP* and *ORRM3* co-silencing construct. The correlation was calculated with 656 points: 618 mitochondrial sites and 38 plastid sites.

**Supplementary Figure S3.** ORRM2 does not interact with RIP1, RIP3 or MEF1. (**A**) ORRM2 does not interact with RIP1 or RIP3. (**B**) ORRM2 does not interact with MEF1 though editing at site *nad7* C963 is reduced in both *ORRM2-*silenced plants and MEF1 mutant plants. EM, yeasts transformed with a vector carrying an empty GW cassette as a negative control.

**Supplementary Table S1.** Primers used in this study

**Supplementary Table S2.** Sites showing a significant reduction of editing extent in ORRM2-silenced sites

**Supplementary Table S3.** Sites showing a significant reduction of editing extent in ORRM3-silenced sites

**Supplementary Table S4.** Plastid editing extent in ORRM2-silenced sites

**Supplementary Table S5.** Plastid editing extent in ORRM3-silenced sites

**Supplementary Table S6.** Effect of RIP1 or RIP3 mutation on sites showing a significant reduction of editing extent in *ORRM3*-silenced sites

**Supplementary Dataset S1.** Number of reads at each editing site (gene-position) for each library (genotype) and probability of observed number of T reads at each editing site
